# Uncovering Novel Plasma Membrane Carboxylate Transporters in the Yeast *Cyberlindnera jadinii*

**DOI:** 10.3390/jof8010051

**Published:** 2022-01-05

**Authors:** Maria Sousa-Silva, Pedro Soares, João Alves, Daniel Vieira, Margarida Casal, Isabel Soares-Silva

**Affiliations:** 1 Centre of Molecular and Environmental Biology (CBMA), Department of Biology, University of Minho, Campus de Gualtar, 4710-057 Braga, Portugal; m.silva@bio.uminho.pt (M.S.-S.); pedrosoares@bio.uminho.pt (P.S.); pg33678@alunos.uminho.pt (J.A.); jdanav@gmail.com (D.V.); mcasal@bio.uminho.pt (M.C.); 2 Institute of Science and Innovation for Bio-Sustainability (IB-S), University of Minho, 4710-057 Braga, Portugal

**Keywords:** *Cyberlindnera jadinii*, carboxylic acids, plasma membrane transporters, phylogeny, protein structure prediction, molecular docking

## Abstract

The yeast *Cyberlindnera jadinii* has great potential in the biotechnology industry due to its ability to produce a variety of compounds of interest, including carboxylic acids. In this work, we identified genes encoding carboxylate transporters from this yeast species. The functional characterization of sixteen plasma membrane carboxylate transporters belonging to the AceTr, SHS, TDT, MCT, SSS, and DASS families was performed by heterologous expression in *Saccharomyces cerevisiae*. The newly identified *C. jadinii* transporters present specificity for mono-, di-, and tricarboxylates. The transporters CjAto5, CjJen6, CjSlc5, and CjSlc13-1 display the broadest substrate specificity; CjAto2 accepts mono- and dicarboxylates; and CjAto1,3,4, CjJen1-5, CjSlc16, and CjSlc13-2 are specific for monocarboxylic acids. A detailed characterization of these transporters, including phylogenetic reconstruction, 3D structure prediction, and molecular docking analysis is presented here. The properties presented by these transporters make them interesting targets to be explored as organic acid exporters in microbial cell factories.

## 1. Introduction

The yeast *Cyberlindnera jadinii*, an Ascomycota belonging to Phaffomycetaceae [1], is an attractive platform for industrial utilization due to its intrinsic robust fermentation features and ability to utilize a wide range of substrates, including hexoses, pentoses, amino acids, and carboxylic acids [2,3,4]. Membrane transporters can be powerful tools used to improve the production of valuable chemical compounds using microbial cell factories [5]. With few exceptions, neither the substrates nor products of cell metabolism can freely cross the plasma membrane. Instead, these molecules require the activity of membrane transporters to cross the cell membrane, both in and out. A unique transporter can recognize distinct substrates, and multiple transporters may be associated with the permeation of a particular substrate across cell membranes [6,7]. The most relevant importers and exporters used for the improvement of microbial cell factories’ production of carboxylic acids were recently reviewed [5]. The Sialate:H^+^ symporter (SHS) family (TC 2.A.1.12) members, where the ScJen1 of *S. cerevisiae* the first monocarboxylate:H^+^ symporter transporter identified in fungi [8] is found, are able to transport carboxylates and sugar acids [5]. The acetate uptake transporter (AceTr) family (TC 2.A.96) includes both importers and exporters of carboxylic acids [9,10], such as the *S. cerevisiae* acetate–propionate–lactate–formate transporter Ato1 (also known as Ady2) [11]. The crystal structures of the bacterial homolog SatP, from *Escherichia coli* and *Citrobacter koseri* [12,13], have revealed three conserved hydrophobic residues (F17–Y72–L131), located in the narrowest constriction site, that intimately contribute to the substrate selectivity of these AceTr members [14,15]. Another well-recognized carboxylate transporter family is the tellurite-resistance/dicarboxylate transporter (TDT) (TC 2.A.16), which, as suggested by its nomenclature, is mainly associated with the uptake of dicarboxylates [16]. The solute carrier (SLC) superfamily [17,18] distributed throughout all domains of life, includes the monocarboxylate transporter (MCT)/SLC16 family (TC 2.A.1.13) found in *Homo sapiens* and the divalent anion:Na^+^ symporter (DASS)/SLC13 family (TC 2.A.47) which can either import or export mono-, di-, and tricarboxylates [19,20,21]. SLC5 members from the solute/sodium symporter (SSS) family (TC 2.A.21) possess a solute-binding domain associated with the transport of small solutes, such as sugars, vitamins, amino acids, monocarboxylates. and smaller organic ions [22].

In the 1980s, distinct plasma membrane organic acids transporters were physiologically characterized in *C. jadinii* [23,24,25,26]: (i) a proton-symporter accepting the monocarboxylates L-lactate and D-lactate, pyruvate, propionate, and acetate [24]; (ii) a dicarboxylate proton-symporter for L-malate, succinate, fumarate, oxaloacetate, and α-ketoglutarate [25,26]; (iii) a tricarboxylate proton-symporter for citrate and isocitrate [23]; and (iv) a low-affinity general permease for the facilitated diffusion of amino acids, and mono-, di- and tricarboxylic acids [23]. The genes encoding these transporters remained unidentified until now. Hence, when searching the *C. jadinii* genome for carboxylic acid transporters homologs, sixteen proteins belonging to the above-mentioned families (e.g., SHS, AceTr, MCT, DASS, SSS, and TDT) were found. The functional characterization of these proteins was achieved via the heterologous expression of their genes in *S. cerevisiae*. To deepen our knowledge and better our understanding of their functional role, a phylogenetic and an *in silico* structural 3D analysis was also performed. Together, these data bring novel insights into *C. jadinii*’s robust physiology, shedding light on the mechanisms of solute plasma membrane transport in this yeast species.

## 2. Materials and Methods

### 2.1. Yeast Cultivation

The yeast strains used in this study are listed in Table 1. The *S. cerevisiae* W303-1A *jen1*Δ *ady2*Δ [27] and *S. cerevisiae* IMX1000 strains [28], lacking carboxylate uptake under the tested conditions, were used to express putative carboxylate transporters. Cultures were maintained at 30 °C on a YPD medium, yeast extract (peptone (1%, *w*/*v*), glucose (2%, *w*/*v*), and agar (2%, *w*/*v*)), or minimal media with the required supplements for growth of strains with auxotrophies. Yeast cells were grown in YNB medium -Difco yeast nitrogen base 0.67%, *w*/*v* (BD Life Sciences, MD, USA) enriched with adequate requirements for prototrophic growth (60 µg/mL of leucine and 40 µg/mL of adenine). The carbon sources used were glucose (2%, *w/v*), acetic acid (0.5%, *v*/*v*, pH 6.0), lactic acid (0.5%, *v*/*v*, pH 5.0), pyruvic acid (0.5% *w/v*, pH 5.0), fumaric acid (1%, *w/v*, pH 5.0), succinic acid (1%, *w*/*v*, pH 5.0), malic acid (1%, *w/v*, pH 5.0), and citric acid (1%, *w/v*, pH 5.5). For spot assays, cells were grown on YNB Glucose without uracil (YNB-Ura) media until mid-exponential phase and adjusted to an OD_640nm_ of 0.1. A set of three 1:10 serial dilutions was created, and 3 μL of each suspension were spotted in the desired media using YNB Glucose-Ura as a control. Cells were incubated at 18 °C for 22 days. At this temperature, the simple diffusion of solutes across the plasma membrane is drastically reduced so that growth on carboxylic acids as the sole carbon and energy source is directly dependent on a functional transporter [27]. For transport assays, cells exponentially growing on YNB Glucose-Ura were harvested, washed twice, and cultivated for 4 h into fresh YNB media supplemented with the suitable carbon source.

### 2.2. Transport Assays

Transport assays were performed as previously described [33]. Cells were incubated 2 min at 30 °C. After 15 or 30 s of incubation with the radiolabeled substrate, the reaction was stopped by adding 100 μL of a cold solution of the tested non-labelled substrate 100-fold concentrated. The suspension was centrifuged for 7 min at 13,200 rpm at 4 °C. The supernatant was carefully rejected, and the pellet was resuspended in 1 mL of deionized cold water and then centrifuged for 10 min at 13,200 rpm at 4 °C. The used labelled carboxylates were [1-^14^C] acetic acid (Perkin Elmer, Waltham, MA, USA); L-[U-^14^C] lactic acid (Perkin Helmer, Waltham, MA, USA); and [2,3-^14^C] succinic acid (Moravek Biochemicals, Brea, CA, USA) and [1,5-^14^C] citric acid (Perkin Elmer, Waltham, MA, USA). Working solutions presented a specific activity ranging from 300 to 4000 dpm depending on the final carboxylate concentrations. Radioactivity was measured in a Packard Tri-Carb 2200 CA liquid scintillation spectrophotometer, with dpm correction. The best-fitting for initial uptake rates was determined through computer-assisted non-linear regression analysis, performed with GraphPad Prism (San Diego, CA, USA) version 4.0 for Windows. The values for kinetic parameters were obtained with a significance level *p* < 0.05. The data shown are mean values of at least three independent experiments, with three replicas each.

### 2.3. Identification, Cloning and Expression of Heterologous Genes

BLASTp analysis revealed six ORFs homologous to the *JEN1* of *S. cerevisiae* (*CEP23088p* (CjJen1), *CEP21966p* (CjJen2), *CEP22358p* (CjJen3), *CEP21989p* (CjJen4), *CEP21602p* (CjJen5), and *CEP25129p* (CjJen6)), five ORFs homologous to the *ATO1* of *S. cerevisiae* (XP_020070445.1 (CjAto1), XP_020073179.1 (CjAto2), XP_020073031.1 (CjAto3), XP_020073178.1 (CjAto4), and XP_020067765.1 (CjAto5)), and other homologs of known carboxylate transporters identified through the presence of specific conserved domains, namely CjSlc16 (XP_020067635.1), CjSlc5 (XP_020068154.1), CjSlc13-1 (XP_020069270.1), CjSlc13-2 (XP_020073044.1), and CjTDT (XP_020068891.1) (Table S1). The sixteen target genes selected from BLASTp searches were amplified by PCR with proofreading polymerase ACCUZYME Mix DNA (Bioline, London, UK) [34] using the genomic DNA extracted from the yeast *Cyberlindnera jadinii* DSM 2361 [35] and cloned in the centromeric plasmid p416GPD [34] under the control of a GPD constitutive promoter (pCj-gene plasmids) according to standard protocols [36]. The *S. cerevisiae* strains W303-1A *jen1Δ ato1Δ* and IMX1000 were used as expression hosts [27,28]. The primers (Eurofins Genomics, Ebersberg, Germany) used for the amplification and cloning of Cj-genes contain restriction sites for *BamH*I and *EcoR*I (CjAto1-4, CjJen1, CjJen3, and CjJen5), *EcoR*I and *Hind*III (CjJen2 and CjJen4), *Xba*I and *EcoR*I (CjJen6), *Spe*I and *Sal*I (CjSlc16, CjSlc5, CjTDT, and CjSlc13-1 genes), *Spe*I and *Xho*I (Slc13-2 gene), and *Spe*I and *EcoR*I (CjAto5) in the forward and reverse primers, respectively (Table S2). The final products were inserted in the p416GPD vector, previously digested with the same restriction enzymes (Thermo Fisher Scientific, Waltham, MA, USA), and are listed in Table 2.

### 2.4. Sequence Alignment and Topology Prediction

Sequence alignment was performed with EMBOSS needle (https://www.ebi.ac.uk/Tools/psa/emboss_needle/) and ClustalOmega (https://www.ebi.ac.uk/Tools/msa/clustalo/) through pairwise sequence alignment. The TMHMM server (http://www.cbs.dtu.dk/services/TMHMM-2.0/) was used for topology prediction of transmembrane segments (TMS). Multiple sequence alignment was also performed with M-Coffee (http://tcoffee.crg.cat/apps/tcoffee/do:mcoffee) for the further validation of identified TMS regions across the protein sequences [37].

### 2.5. Three-Dimensional Modelling, Molecular Docking Studies and Pore Radius Simulations

Molecular docking simulations were performed as previously described [33]. Ligand structures of acetic, lactic, succinic, and citric acids were downloaded from the Zinc database [38]. Only deprotonated forms of each acid were used in the docking prediction, with the protonation states adjusted to match a pH of 5.0–6.0. The 3D substrate structures were built by inputting canonical SMILES (Simplified Molecular Input Line Entry Specification) strings into UCSF Chimera [39], being minimized before molecular docking in PyRx software [40] using AutoDock Vina. The simulated interactions were analyzed in 2D and 3D pose views using both Chimera and Maestro version 11.2 (Schrödinger, Inc. New York, NY, USA). The HOLE program (2.2.005 Linux) was used to predict the pore radius throughout each studied transporter [41]. The radiuses of the homolog transporters were compared in a graph, with the coordinate in the direction of the channel vector serving as the x-axis. The images of the predicted pores were obtained using Visual Molecular Dynamics program (VMD, 1.9.3) [42].

### 2.6. Phylogenetic Reconstructions

A total of over 10,000 inferred proteomes from the NCBI Assembly platform, including the different domains of the Tree of Life, were downloaded as individual FASTA files (all belonging to the RefSeq subsection of NCBI) and converted into a local database. To avoid redundancies, only sequences from a single genome of a given species were considered. In these cases, the specimen with the higher number of proteins described in the database was selected. A BLAST search, with a cut-off e-value 10^−10^ and an associated query-cover value higher than 65%, was performed on our in-house database using twelve queries (5 CjATO, 6 CjJEN, and CjSLC5), corresponding to previously detected carboxylate transporters on the proteome of *C. jadinii*. The results from analysis of the five Ato1 homologs were merged into a single file by removing redundant proteins across results. The same approach was taken for the six queries of Jen1 homologs in *C. jadinii*. The CjSlc5 (XP_020068154.1) sequence was individually used as a query for the search of homologs of each protein. Given the fact that a detailed phylogeny of Ato1 was recently published [10], the analysis only included Ascomycota. Retrieved protein sequences were aligned using the MAFFT online server [43], which incorporates multiple alignment strategies. Sequences that were not extensively matching across the conserved region of the alignment were further excluded from the phylogenetic analysis. These sequences, in many cases, could represent a lower quality stretch of the genome where they are located or the incomplete annotation of the full gene, and they should not necessarily be regarded as non-functional genes. A phylogenetic reconstruction was performed using Maximum likelihood, more appropriate for the deeper divergences under analysis here, using MEGA7 [44] and the Jones–Taylor–Thornton (JTT) substitution model. Bootstrap was performed for 1000 repetitions. Obtained phylogenetic trees were displayed and edited in FigTree v.1.4.4. (http://tree.bio.ed.ac.uk/).

## 3. Results

### 3.1. Characterization of Carboxylic Acid Transport Systems in Cyberlindnera jadinii

**Figure 1:**
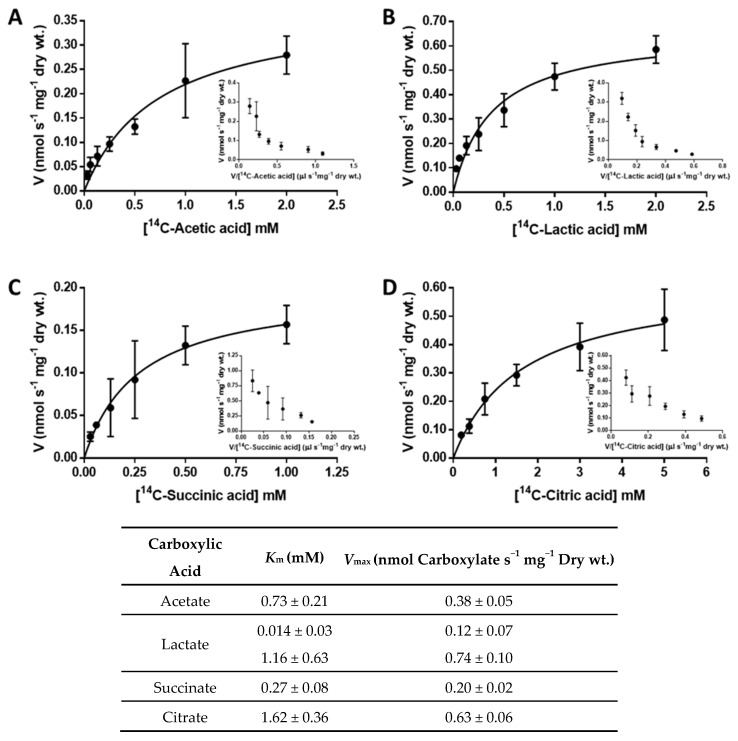
Initial uptake rates of labelled (**A**) ^14^C−acetic acid, pH 6.0; (**B**) ^14^C−lactic acid, pH 5.0; (**C**) ^14^C−succinic acid, pH 5.0; and (**D**) ^14^C−citric acid, pH 5.5, as a function of the acid concentration in *C. jadinii* DSM 2361 cells. The inserts represent the Eadie–Hofstee plots of the data presented in the respective main chart. The data shown are mean values of at least three independent experiments, and the error bars represent the standard deviation. The bottom-table presents the best fitting transport kinetic values for carboxylate transport systems in *C. jadinii* DSM 2361 cells, estimated with computer-assisted non-linear regression analysis.

Cells of *Cyberlindnera jadinii* DSM 2361 grown on acetic (0.5%, *v*/*v*; at pH 6.0), lactic (0.5%, *v*/*v*; at pH 5.0), succinic (1.0%, *w*/*v*; at pH 5.0), and citric (1.0%, *w*/*v*; at pH 5.5) acids displayed the ability to transport these substrates through a Michaelis–Menten kinetics (Figure 1), an indication of the presence of mediated transport for mono-, di-, and tricarboxylic acids in this strain [45]. A non-linear regression analysis of the initial uptake rates of each acid allowed for the estimation of the kinetic parameters of these transport systems (Figure 1).

### 3.2. Heterologous Expression of Cyberlindnera jadinii Genes Encoding Putative Transporters in Saccharomyces cerevisiae

Currently, there are four *C. jadinii* genome sequences at NCBI: three from the strain CBS 1600/NRRL Y-1542 and one from the NBRC0988 (DSM 2361) strain. Here, the CBS 1600/NRRL Y-1542 genomes were screened for paralogs of ScAto1 and ScJen1 transporters from *S. cerevisiae*, resulting in the identification of five ScAto1 (CjAto1-5) and six ScJen1 (CjJen1-6) homologs. In parallel, we performed an analysis for homology against conserved domains from functionally characterized carboxylic acid transporters families. This second strategy enabled the detection of a separate set of genes belonging to the families SSS (CjSlc5), MCT (CjSlc16), DASS (CjSlc13-1 and CjSlc13-2), and TDT (CjTDT), which are also candidates for encoding carboxylate transporters.

**Figure 2:**
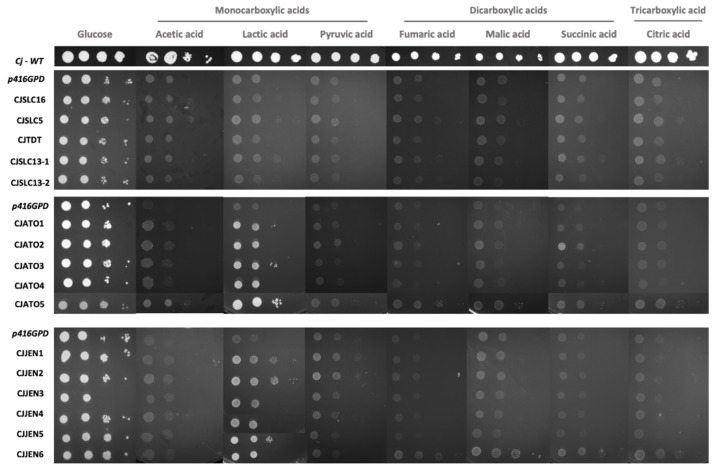
Functional analysis of sixteen *C. jadinii* carboxylate transporters in *S. cerevisiae* IMX1000 based on their ability to grow on YNB-URA media containing glucose (2% *w*/*v*), acetic acid (0.5% *v*/*v*; pH 6.0), lactic acid (0.5% *v*/*v*; pH 5.0), pyruvic acid (0.5% *w/v*; pH 5.0), fumaric acid (1% *w*/*v*; pH 5.0), malic acid (1% *w*/*v*; pH 5.0), succinic acid (1% *w*/*v*; pH 5.0). or citric acid (1% *w*/*v*; pH 5.5) as the sole carbon and energy source. The *C. jadinii* DSM 2361 strain was included as a positive control, and *S. cerevisiae* IMX1000 cells transformed with p416GPD were used as a negative control. Cells were grown at 18 °C for 22 days.

The *S. cerevisiae* IMX1000 strain was transformed with each of the sixteen putative *C. jadinii* carboxylate transporters cloned in the plasmid p416GPD [34], a centromeric plasmid with a strong constitutive promoter. Transformants were evaluated regarding their ability to grow on carboxylic acids as sole carbon and energy sources (Figure 2). The positive control cells of *C. jadinii* DSM 2361 presented a noticeable growth on the tested carbon sources when compared to *S. cerevisiae* transformant strains (Figure 2). All of the *S.* *cerevisiae* transformants were able to grow on acetic acid except for cells expressing *CjTDT*. The strains expressing the *CjATO1-5* homologs recovered the ability to grow on lactic acid. Furthermore, cells expressing *CjATO2* also displayed improved growth on pyruvic, malic, and succinic acid. The expression of *CjATO5* allowed cells to grow on all tested mono-, di-, and tricarboxylic acids. The cells expressing *CjJEN1-6* displayed improved growth on monocarboxylic acids. The expression of *CjJEN6* improved cells’ growth on dicarboxylic and tricarboxylic acids. The expression of *CjSLC5* and *CjSLC13-1* improved growth on all tested carbon sources, although to a lesser extent than *CjATO5* and *CjJEN6*. Cells expressing *CjSLC13-2* presented slightly decreased growth on lactic and citric acid when compared to the negative control. The expression of *CjTDT* did not allow for improved growth in any of the acids (Figure 2). Equivalent results were obtained using the *S. cerevisiae jen1*Δ *ato1*Δ strain expressing each of the sixteen putative *C. jadinii* carboxylate transporters (not shown). Protein expression levels were not determined because further studies demonstrated the presence of transporter activity for all the putative carboxylate transporters.

### 3.3. Transport Assays of Mono-, Di-, and Tricarboxylates

**Figure 3:**
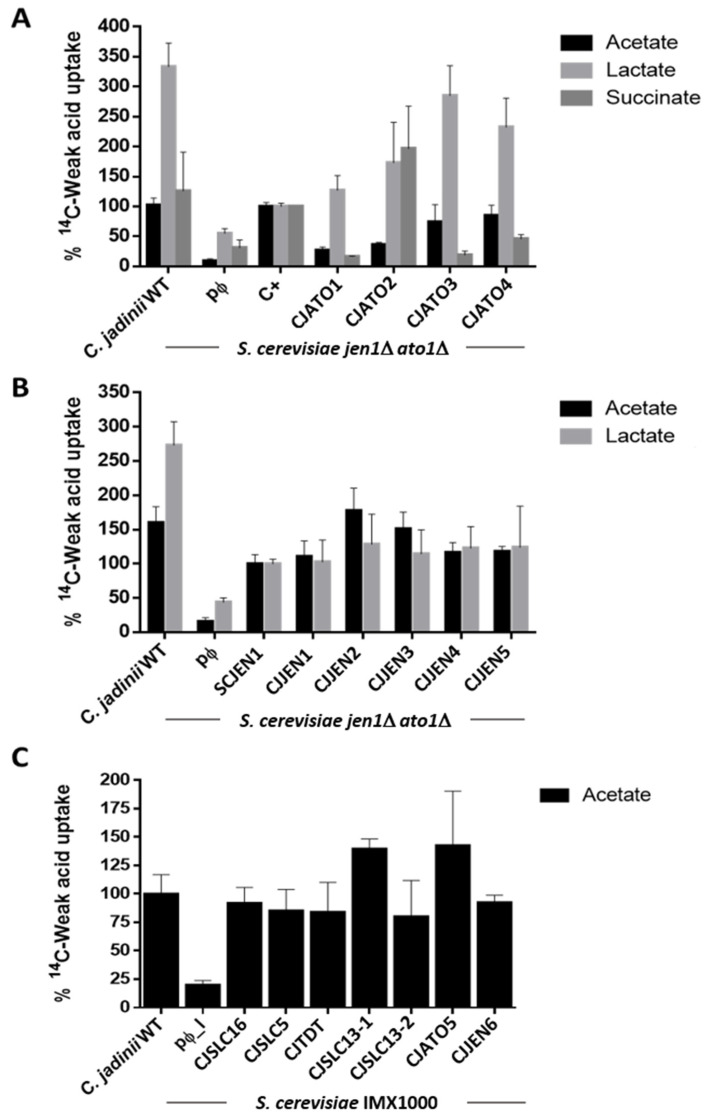
Transport of radiolabeled carboxylates in *C. jadinii* DSM2361 strain (**A**–**C**), *S. cerevisiae jen1*Δ *ato1*Δ (**A**,**B**), and IMX1000 (**C**) cells expressing the *C. jadinii CjATO1-5, CjJEN1-6, CjSLC16, CjSLC5, CjTDT, CjSLC13-1,* and *CjSLC13-2* proteins, as well as an empty vector (pɸ), as negative control. (**A**) Uptake of 1 mM of ^14^C-acetic acid (pH 6.0), ^14^C-lactic acid (pH 5.0), and ^14^C-succinic (pH 5.0) acid. For acetic and lactic acid uptake, a value of 100% (C+) corresponds to the uptake in *S. cerevisiae* cells transformed with the plasmid p416GPD-expressing genes encoding the native transporters *ATO1* and *JEN1*, respectively. For succinic acid, a value of 100% (C+) corresponds to isogenic cells expressing the *Candida albicans JEN2* transporter cloned in the p416GPD plasmid. (**B**) Uptake of 1 mM ^14^C-acetic acid (pH 6.0) and 1 mM ^14^C- lactic acid (pH 5.0). A value of 100% corresponds to the uptake of acetic and lactic acid displayed by cells expressing the *JEN1* transporter. (**C**) Uptake of 1 mM of ^14^C-acetic acid (pH 6.0). A value 100% corresponds to the uptake of acetic acid by *C. jadinii* DSM 2361 cells. Cells were grown on YNB Glucose, washed, and incubated on YNB containing the respective carbon source (see materials and methods) used in the uptake assay for 6 h (acetate 0.5%, pH 6.0) or 5 h (lactate 0.5%, pH 5.0; succinate 1%, pH 5.0).

To functionally validate the sixteen genes of interest as carboxylate transporters, we used cells of *S. cerevisiae* W303-1A *jen1*Δ *ato1*Δ and IMX1000 strains individually expressing the sixteen Cj-transporters to evaluate uptake of [^14^C]acetic acid (pH 6.0), [^14^C]lactic acid and [2,3-^14^C]succinic acid (pH 5.0) (Figure 3). Cells expressing *CjATO1-4* and *CjJEN1-5* presented increased uptakes for labelled acetate and lactate when compared to the strain carrying the empty vector, supporting their role as acetate and lactate transporters (Figure 3A,B). For acetate uptake, the values obtained for CjAto3 and CjAto4 were similar to that of native ScAto1, while for lactate, higher values were found in comparison to ScJen1, the main lactate transporter of *S. cerevisiae*. Likewise, CjJen2, CjJen3, and CjJen6 displayed superior acetate uptake when compared to ScJen1 (Figure 3B,C), though lactate uptake was identical across CjJen1-6. Cells expressing *CjATO2* presented a higher uptake for succinate than those expressing *CaJEN2*, the dicarboxylate transporter from *Candida albicans* that was used as a positive control in the assay (Figure 3A).

We then performed a more detailed analysis of genes displaying transport activity. First, the kinetic parameters of monocarboxylic acid uptake were estimated in the *S. cerevisiae* W303-1A *jen1*Δ *ato1*Δ strain expressing CjATO1-4 and CjJEN1-5 (Table 3). The transporters presented *V*_max_ values ranging from 0.49 to 4.92 nmol acetate s^−1^ mg^−1^ dry wt and *K*_m_ values ranging from 1.26 to 12.18 mM acetate. Succinate transport activity was found to associated with the expression of *CjJEN6, CjSLC5, CjATO5,* and *CjATO2*; the estimated velocities (*V*) for the uptake of succinate 1.0 mM (pH 5.0) were 0.131, 0.240, 0.277, and 0.282 nmol succinate s^−1^ mg^−1^ dry wt., respectively; for the empty vector, a residual value of 0.039 nmol succinate s^−1^ mg^−1^ dry wt. was determined in cells transformed with the empty vector.

**Figure 4:**
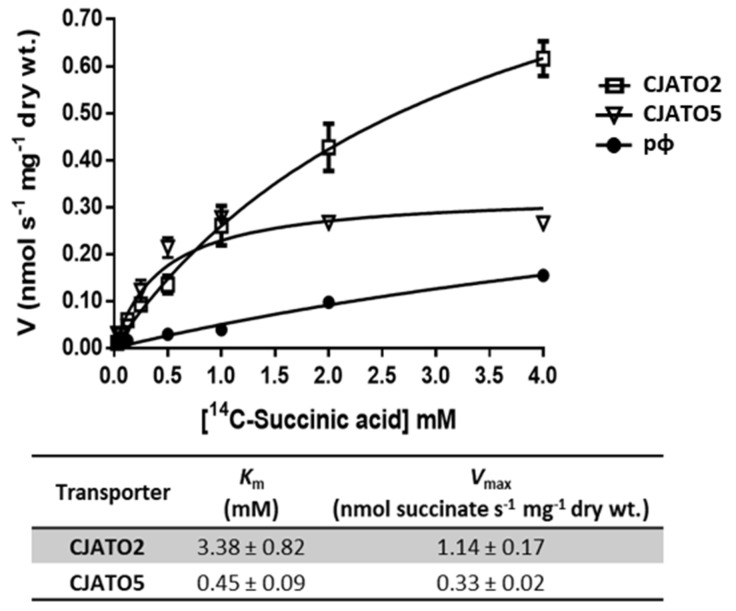
Initial uptake rates of radiolabeled ^14^C−succinic acid, pH 5.0, as a function of the acid concentration in *S. cerevisiae* IMX1000 cells expressing the pCjAto2 (empty squares) and pCjAto5 (empty triangles). As a negative control, cells transformed with the p416GPD were used (full circles). The kinetic parameters presented in the table were estimated using the computer-assisted non-linear regression analysis of the plots.

The *S. cerevisiae* IMX1000 strain, deleted in 25 genes coding for all known and putative carboxylate transporters, was used for the heterologous expression of *CjSLC16, CJSLC5, CjSLC13-1, CJSLC13-2, CjTDT, CjATO5,* and *CjJEN6*. All transformants presented activity for acetate uptake (Figure 3C), pointing to the role of these proteins as carboxylate transporters. The kinetic parameters for labelled lactic and succinic acid in cells expressing *CjATO2* were the following: *K*_m_ 4.33 ± 0.70 mM lactate, *V*_max_ 1.69 ± 0.17 nmol lactate s^−1^ mg^−1^ dry wt., *K*_m_ 3.38 ± 0.82 mM succinate, and *V*_max_ 1.14 ± 0.17 nmol succinate s^−1^ mg^−1^ dry wt. at pH 5.0 (Figure 4). The initial uptake rates of labelled succinic acid estimated in cells transformed with pCjAto5 also revealed Michaelis–Menten kinetics with the following parameters: *K*_m_ = 0.45 ± 0.09 mM and *V*_max_ = 0.33 ± 0.02 nmol s^−1^ mg^−1^ dry wt. (Figure 4). Cells transformed with the empty vector presented a first order kinetics associated with the diffusion of the acid (Figure 4) [33].

**Figure 5:**
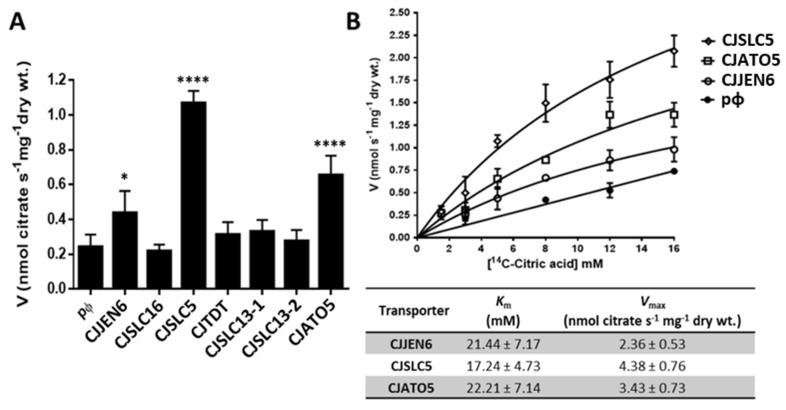
Citrate transport (pH 5.5, 30 °C) in *S. cerevisiae* IMX1000 cells expressing *C. jadinii* transporters. (**A**) Uptake of ^14^C-citric acid 5 mM in cells expressing the transporters *CjJEN6, CjSLC16, CjSLC5, CjTDT, CjSLC13-1, CjSLC13-2,* and *CjATO5,* as well as those transformed with empty vector (pɸ). Statistical significance was estimated using one-way ANOVA followed by a post hoc Tukey’s multiple comparison test as follows: * *p* < 0.1 and **** *p* < 0.0001, citrate uptake significantly different from cells transformed with the empty plasmid. (**B**) Initial uptake rates of radiolabeled ^14^C-citric acid as a function of the acid concentration in cells expressing CjJen6, CjSlc5, and CjAto5; the table summarizes the kinetic parameters estimated with the computer-assisted non-linear regression analysis of the plots.

Citrate uptake was evaluated in the *S. cerevisiae* IMX1000 strains expressing *CjJEN6, CjSLC16, CjSLC5, CjTDT, CjSLC13-1, CjSLC13-2,* and *CjATO5* (Figure 5A). The kinetic parameters of citrate uptake by the most promising citrate transporters CjAto5, CjJen6, and CjSlc5 are presented in Figure 5B. The transporter with the highest activity was CjSlc5, followed by CjAto5. Despite having the lowest transport activity, CjJen6 still allowed for the growth of *S. cerevisiae* cells on media containing citrate as the sole carbon and energy source (Figure 2).

### 3.4. In Silico Structural Analysis of the CjAto2 and CjAto5 Transporters

To understand the structural features of the found novel dicarboxylate and tricarboxylate transporters, the CjAto2 and CjAto5 transporters were analyzed using three approaches: (i) the determination of the conserved residues found in the multiple alignment of functionally characterized homologs (Figure S1), (ii) 3D structure prediction (Table S3), and (iii) the molecular docking of substrates (Tables S4 and S5).

The signature NPAPLGL(M/S) motif [10] of the AceTr family, crucial for substrate uptake, presented some interesting substitutions among CjAto transporters (Figure S1). The highly conserved L93-ScAto1 was replaced by the residues I75 and M85 in CjAto2 and CjAto5, respectively. The 3D structure of *Citrobacter koseri* Ck_SatP revealed that the central and narrowest hydrophobic constriction of the anion pathway of the channel is formed by F17-Y72-L131 residues [12]. This narrowest constriction in ScAto1 corresponds to F98-Y155-L219 [14,15]. The equivalent F98 residue was found to be conserved in the CjAto1-5 homologs. However, in CjAto5, two relevant substitutions are found: the equivalent Y155 was replaced by F147 and the equivalent L219 was replaced by V210.

**Figure 6:**
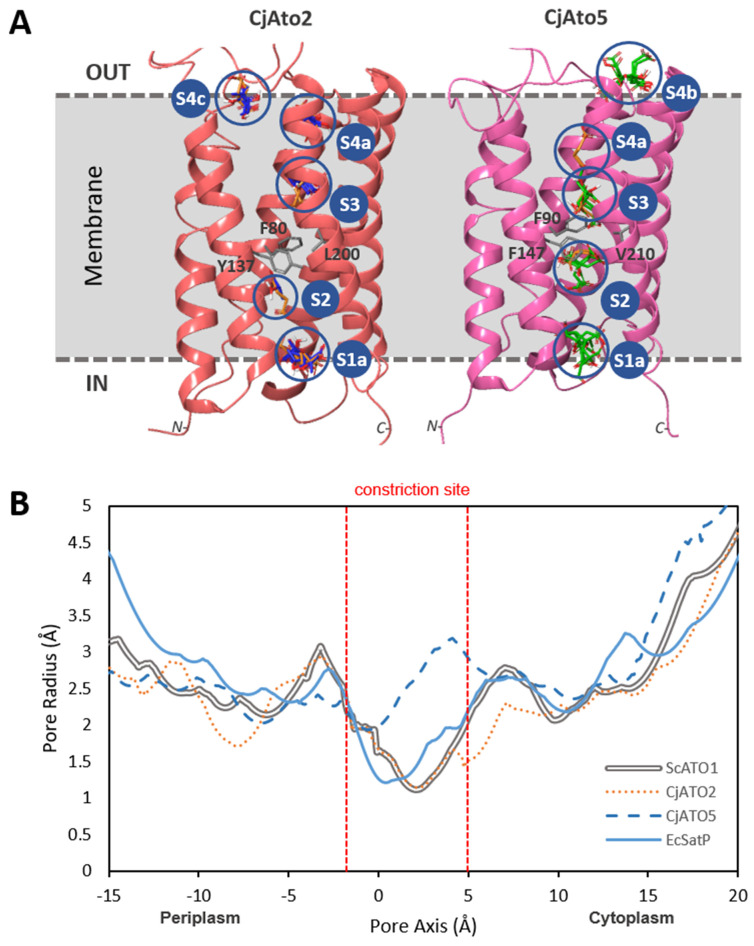
Predicted 3D structure of CjAto2 and CjAto5 transporters. (**A**) Molecular docking of CjAto2 and CjAto5 3D models based on SatP_Ck structure (PDB 5YS3) with the substrates lactate (blue ligand), succinate (orange ligand), and citrate (green ligand). The four binding sites (S1−4) described in SatP_Ck were also found in CjAto2 and CjAto5. The localization of N− and C-terminal of the proteins is depicted. (**B**) Simulations for the pore radius profiles through the channel axis in the ScAto1 (grey line), CjAto2 (orange dotted line), CjAto5 (dark blue dashed-line), and EcSatP (blue line) proteins. The core of the channel, where the constriction site was found, is delimited by the vertical dashed red lines.

The predicted 3D structure of CjAto2 and CjAto5 were obtained by homology threading using the crystal structure of the bacterial homolog Ck_SatP (PDB 5YS3) [12] as a template (Figure 6). Substrate docking studies for lactate, succinate, and citrate identified the residues involved in substrate binding in these predicted structures (Table S4). Deprotonated succinate (mono- and dianionic) presented strong interactions with residues S211 (end of TMS5) and Q219 (beginning of TMS6) in CjAto2, two residues not conserved among homologs of this family (Table S4). Furthermore, in CjAto2, a higher number of strong interactions for succinate were detected when using ScAto1 as reference, which does not transport this acid.

For CjAto5, lactate is likely to interact with Q103 (end of TMS1), G220 (end of TMS5), and T229 and K230 (both in the beginning of TMS6), two residues that have also been predicted to interact with citrate. For succinate, it is predicted to interact with K221 (end of TMS5) and E125 (located in TMS2). This latter residue is also predicted to interact with citrate, together with residues Q103 and K230. Interestingly, Q103 and K230 are two amino acids residues quite unique at this position, as they were not found in the remaining functionally characterized AceTr members, which instead presented conserved arginine in the majority of these positions (Figure S1).

The binding affinities of the residues presenting strong Van der Waals interactions with the substrates were analyzed (Table S5). Similar to the Ck_SatP crystal structure [12], four binding sites were predicted through the channel axis in all CjAto proteins (Figure 6 and Figure S2). Representative results for CjAto2 and CjAto5 are presented in Figure 6A. The estimated lactate binding affinity values were found to be similar in ScAto1 and CjAto2. As for succinate, binding affinity values for CjAto2 and CjAto5 were higher than native Ato1 on the S2-binding site (Figure 6; Table S5). An increased citrate binding affinity was found in the S2 binding site of CjAto5 (residue K221) (Table S5).

The pore radius at the FYL-constriction site is shown in Figure 6B for CjAto2 and CjAto5. A significant increase of the pore radius was found in CjAto5, where the narrowest site was of 2.1 Å, compared to the 1.11 Å present in ScAto1. This could lead to a structural alteration in the channel pore constriction site, thus allowing for the passage of larger molecules such as citrate. In CjAto2, the constriction pore size was found to be similar to ScAto1 (Figure 6 and Figure S3), although it is able to transport larger molecules such as succinate.

### 3.5. In Silico Structural Analysis of the CjJen5 and CjJen6 Transporters

Previous studies have identified ScJen1 motifs involved in the substrate translocation pathway [27,46]. The analysis of the multiple alignment of functionally characterized homologs revealed that the conserved motif ^379^NXX[S/T]HX[S/T]QD^387^, located at TMS7, and residues Q498 and N501 (TMS11) are conserved in all the CjJen homologs (Figure S4). In TMS5, a conserved S266 residue, whose substitution to leucine abolished CaJen1 activity [47], was found to be replaced by glycine in CjJen3 and CjJen4, although these presented transport activity. The L366 residue, conserved across the CjJen homologs, was found to be substituted by V260 in CjJen6. The residue W154 (TMS1) present in CjJen6, is also present in the dicarboxylate transporters CaJen2 and KlJen2 [31,48]. CjJen5 and CjJen6 proteins share 75% identity, but they present very different substrate specificities.

### 3.6. Phylogenetic Analysis

A phylogenetic analysis of *C. jadinii* carboxylate transporters belonging to the SHS (TC 2.A.1.12) and SSS (TC 2.A.21) transporter families was carried out throughout the Tree of Life. For the AceTr (TC 2.A.96) family, the analysis was limited to Ascomycota, since a broad phylogenetic analysis was already presented in a previous study [10]. Descriptions of the different homologs used in the phylogenetic reconstructions, according to NCBI, are available in Figures S5–S7.

**Figure 7:**
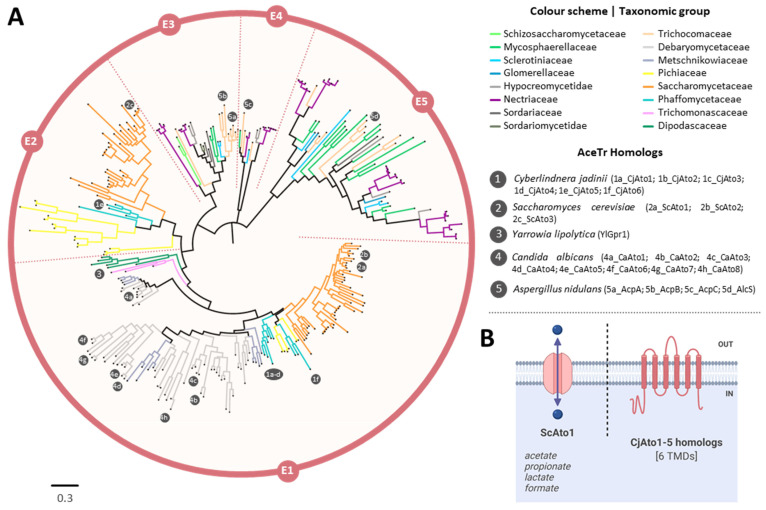
Maximum likelihood phylogenetic tree of AceTr family members (TCDB 2.A.96) present in ascomycetes. (**A**) Branch lengths are proportional to sequence divergence, and different colors correspond to the different families. The clades indicated as E1, E2, E3, E4, and E5 were created to facilitate the tree description in the main text and are not meant to provide any type of classification. Homologs relevant for the discussion through the manuscript are highlighted. (**B**) A simplified scheme of Ato1 native and CjAto proteins with inferred substrate specificities is depicted.

For the AceTr family, a total of 304 hits were obtained for the BLAST search in the NCBI’s Assembly database, using complete genomes only and CjAto1-5 homologs as queries. Eight sequences lacking large conserved regions were excluded for a final dataset of 296 sequences. The phylogenetic tree presents five main clades (E1–E5, Figure 7), depicting the functionally characterized AceTr members described in literature. Clade E1 includes ScAto1 and ScAto2 from *S. cerevisiae* [11], Gpr1 from *Y. lipolytica* [10,49], and the eight Ato homologs from *C. albicans* [50,51]; the *S. cerevisiae* Ato3 is in clade E2 [52]; the acetate transporters AcpA and AcpB from *A. nidulans* belong to clade E3 [53,54]; and the *A. nidulans* AcpC belongs to clade E4 [54] and the AlcS from *A. nidulans* belongs in clade E5 [55], both with unknown function. CjAto1-6 were found into two distinct clades—five were found to be located in E1 and CjAto5 appeared in E2. Half of the AceTr members were found to be located in ScAto1/ScAto2/YlGpr1 clade, the majority belong to the Debaryomycetaceae (*Candida* and *Debaryomyces* species) and Saccharomycetaceae (*Saccharomyces, Kluyveromyces,* and *Lachancea* species) families. Some of these species have homologs in the E2 clade too, probably due to the occurrence of an ancient genetic duplication phenomena, as previously reported [10]. Homologs from the *Fusarium* spp. genus were found to be dispersed through distinct clades.

**Figure 8:**
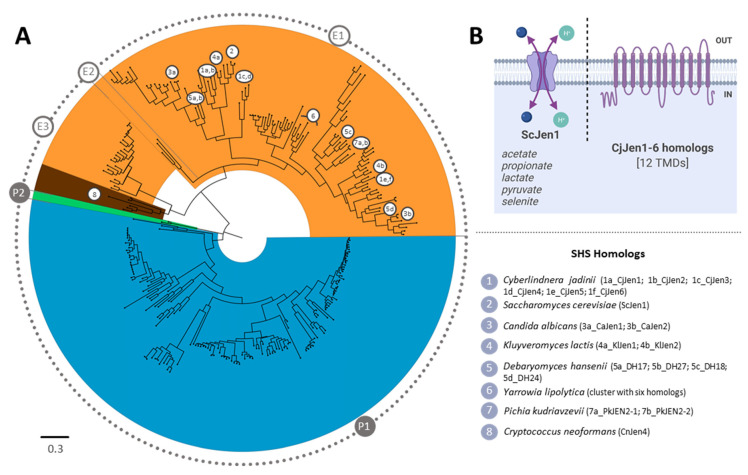
Maximum likelihood phylogenetic tree of SHS family members (TCDB 2.A.1.12). (**A**) Groups indicated as E1, E2, and E3 for eukaryotic clades and P1 and P2 for prokaryotic clades were established to enable the following of the tree description in the main text and are not meant to provide any type of classification. Major taxonomic groups are indicated in shades of blue—bacteria; orange—ascomycetes; brown—basidiomycetes; and green—archaea. Homologs relevant for the discussion through the manuscript are highlighted. (**B**) A simplified scheme of Jen1 native and CjJen proteins with inferred substrate specificities is depicted.

A total of 306 hits belonging to the SHS family were obtained from the BLAST search with the six CjJen members. Fourteen sequences that lacked large portions of the conserved regions were excluded for a final dataset of 292 sequences. The phylogenetic reconstruction proposed a basal split into prokaryotic and eukaryotic organisms, forming two monophyletic clades (Figure 8). The prokaryotic clade gathers two branches, P1 and P2 that showed bacterial and archaeal homologs, respectively. The P1 clade mostly encompasses homologs belonging to Proteobacteria, Acidobacteria, and Actinobacteria phyla. The P2 clade only includes two archaeal homologs belonging to the *Thermoplasma* genus. The eukaryotic clade was shown to split into three branches labelled as the E1, E2, and E3 clades. The E1 clade comprises two subclades: the monocarboxylate transporter Jen1-homologs—namely ScJen1 from *S. cerevisiae* [8], CaJen1 from *C. albicans* [47], KlJen1 from *K. lactis* [48], and two homologs DH17 and DH27 from *D. hansenii* [56] and the dicarboxylate transporter Jen2-homologs—namely CaJen2 from *C. albicans* [31,33], KlJen2 from *K. lactis* [48], DH18 and DH24 from *D. hansenii* [56], the six clustered *Y. lipolytica* homologs characterized as functional mono/dicarboxylate transporters [57], and the PkJen2-1 (a dicarboxylate transporter) and PkJen2-2 (a di- and tricarboxylate transporter) from *Pichia kudriavzevii* [58]. ln the E2 clade, only three homologs were found—one belonging to *Pochonia chlamydosporia,* the other to *Aspergillus fumigatus*, and another to *Zymoseptoria tritici*. The E3 clade was found to contain homologs from Ascomycota and Basidiomycota, namely Jen1 from *Cryptococcus neoformans*, reported as a 3-bromopyruvate transporter [59]. All the CjJen homologs were shown to be in the E1 clade—four in the Jen1-cluster (CjJen1-4), and two (CjJen5,6) in the Jen2-cluster.

The CjJen homologs were found in groups of two, suggesting their origin derived from a recent genetic duplication phenomenon. Previous studies have reported that a precursor form of Jen1p (preJen1) originated form a duplication of an ancestral Jen2 [60]. Only microbial genomes were found in the SHS phylogenetic reconstruction, from which around 60% were shown to belong to eukaryotic taxa.

**Figure 9:**
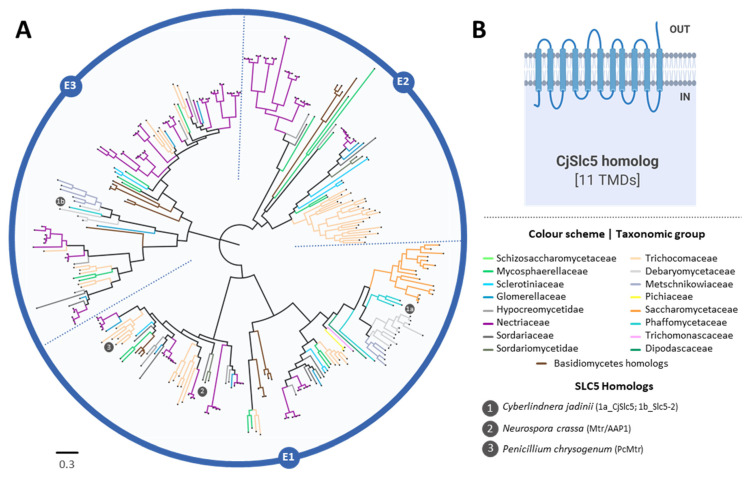
Maximum likelihood phylogenetic tree of SSS (SLC5 homolog) family members (TCDB 2.A.21). (**A**) Branch lengths are proportional to sequence divergence. Groups indicated as E1, E2, and E3 were defined to enable the following of the tree description in the main text and are not meant to provide any type of classification. Homologs relevant for the discussion through the manuscript are highlighted. (**B**) A simplified scheme with predicted topology for CjSlc5 protein is depicted.

The phylogenetic reconstruction of the SSS-tree (SLC5 member) showed a total of 311 hits obtained from the BLAST search. Ten protein sequences that lacked large conserved regions were excluded for a final dataset of 301 sequences (Figure 9). The CjSLC5 is a member of solute carrier superfamilies 5 and 6-like. Members of this superfamily include specific solute-binding domains: SLC5 proteins are also called the sodium/glucose co-transporters or solute sodium symporters, and SLC6 proteins are Na^+^/Cl^−^-dependent transporters and nucleobase-cation-symport-1 (NCS1) transporters. CjSlc5 homologs were only identified in eukaryotic organisms, mostly belonging to the Asco- and Basidiomycota phyla. Homologs were found to be distributed across three major clades, E1, E2, and E3. The E1 clade contains the sole *C. jadinii* homolog, CjSlc5, phylogenetical closer to the homologs from the Metschnikowiaceae, Debaryomycetaceae, and Saccharomycetaceae families. In E1, there is a minor subclade that includes basidiomycetes members from *Cryptococcus*, *Ustilago,* and *Sporisorium* genera, as well as the amino acid transporters NcMtr/AAP1 and PcMtr from *Neurospora crassa* [61] and *Penicillium chrysogenum* [62], respectively. The E2 and E3 clades contain mainly yeast homologs and few basidiomycetes members. Interestingly, SLC5 homologs were found to be present in several *Fusarium* species, which constitute 40% of total members present in the phylogenetic tree together with *Aspergillus* and *Penicillium* species; however, the majority of these remain uncharacterized.

## 4. Discussion

### 4.1. The Cyberlindnera jadinii Carboxylic Acid Transporters Are Functional in Saccharomyces cerevisiae

**Figure 10:**
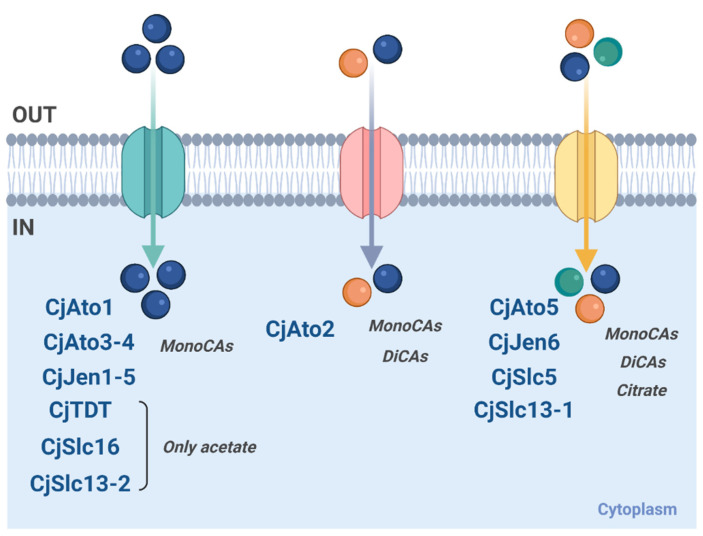
Carboxylic acid transporters from *C. jadinii* functionally characterized by heterologous expression in *S. cerevisiae*. Color circles represent the specificities uncovered for each of the protein-system: monocarboxylic acids—blue-filled circle; dicarboxylic acids—orange-filled circle; tricarboxylic acids—green-filled circle. IN—intracellular space; OUT—extracellular space. Created with biorender.com.

In this study, we characterized sixteen carboxylate transporters in *Cyberlindnera jadinii* belonging to distinct families (Figure 10). The physiological characterization of the *C. jadinii* DSM 2361 strain revealed the existence of mediated transport systems for acetate, lactate, succinate, and citrate. Previous studies with the *C. jadinii* IGC/PYCC 3092 strain [23,24,25] presented different kinetic parameters, which could have been due to the distinct growth conditions used [45]. The genetic background is another source of variation, since the two *C. jadinii* strains with the full genome sequenced presented differences in genome size and the number of predicted proteins [1].

Genome-wide scan approaches were used to uncover transporter proteins via the search for homologs of carboxylate transporters and conserved family domains.

The heterologous expression of *CjATO1-5, CjJEN1-6, CjSLC16, CjSLC5, CjSLC13-1,* and *CjSLC13-2* in *S.* *cerevisiae* strains improved cell growth on carboxylic acids, suggesting their role as carboxylate transporters (Figure 2). The expression of five CjAto transporters encoding genes promoted cell growth in acetic and lactic acid as the sole carbon and energy source. Cells expressing CjAto2 and CjAto5 also presented improved growth on dicarboxylates (malic and succinic acids) and CjAto5 on tricarboxylates (citrate). The bacterial homolog SatP from *Escherichia coli* is the only member from the AceTr family able to transport both monocarboxylates (acetate and lactate) and dicarboxylates (succinate) [63]. In this work, we functionally characterized the first member of the AceTr family able to transport mono-, di-, and tricarboxylic acids. CjAto5 was found to present an affinity for succinate (Figure 4) similar to other high-affinity dicarboxylate transporters, namely CaJen2 from *C. albicans* (*K*_m_ 0.49 mM), DH18 (*K*_m_ 0.31 mM) and DH24 (*K*_m_ 0.16 mM) from *D. hansenii,* and KlJen2 (*K*_m_ 0.11 mM) from *K. lactis* [5,31]. On the other hand, CjAto2 is a low-affinity transporter for succinate, suggesting distinct physiological roles of these transporters regarding succinate permeation across plasma membranes. In view of previous studies [64], CjAto2 might behave as an exporter and CjAto5 as an importer. Recently, Alves et al. (2020) reviewed the evolutionary roots of orthologues of ScAto1 transporters present in twelve *Candida* species, but these were not yet functionally characterized [51]. The monocarboxylate transporters ScAto1 and *Y. lipolytica* Gpr1 are the only AceTr member functionally characterized in yeast [10]. Here, we have described five new yeast AceTr members transporting mono-, di-, and tricarboxylates.

*S. cerevisiae* cells expressing *CjJEN1-6* homologs promoted cell growth on acetic and lactic acid, thus corroborating the specificity already characterized for monocarboxylates (Figure 2) [8,27]. Additionally, cells expressing *CjJEN6* grew in all tested di- and tricarboxylates, a phenotype identical to the one reported in *Y. lipolytica* for the six homologs of ScJen1 found in this species (YALI0B19470g, YALI0C15488g, YALI0C21406g, YALI0D24607g, YALI0D20108g, and YALI0E32901g) [65].

The *C. jadinii* protein homologs members of the SSS (CjSlc5) and DASS (CjSlc13) family revealed to be functional monocarboxylate, dicarboxylate, and tricarboxylate transporters. *S. cerevisiae* cells expressing *CjSLC5* and *CjSLC13-1* presented improved growth in all tested carbon sources (lactic, pyruvic, fumaric, malic, succinic, and citric acid), with the phenotype being more evident in CjSlc5 transformant cells. Transport assays of labelled mono-, di-, and tricarboxylates confirmed the role of CjSlc5 as a carboxylate transporter. The reported function of CjSlc13-1 is also in accordance with data reported for the DASS family SLC13 members presenting affinity for succinate, citrate, and α-ketoglutarate [66,67].

Within this work, several carboxylate transporters were functionally characterized as displaying a broad range of substrate affinities and transport capacities. Further studies on the expression regulation of these genes in *C. jadinii* are needed to fully understand the existence of such high number of transporters. This knowledge will certainly shed light on the well-adapted performance of this species to environments rich in carboxylic acids.

### 4.2. CjSlc5p, CjAto5p and CjJen6 Are Lactate–Succinate–Citrate Transporters

In this work, we have described transporters from three distinct families able to accept mono-, di-, and tricarboxylic acids. The expression of *CjSLC5, CjATO5,* and *CjJEN6* in *S. cerevisiae* enabled cells to grow on all tested acids. The kinetic parameters of citrate uptake (Figure 5) revealed that these three transporters displayed low affinity and high capacity for citrate. Considering the kinetic parameters for citrate uptake previously reported in *C. jadinii*, the gene encoding the high-affinity/low capacity system remains unidentified [23]. Recent studies uncovered three fungal plasma membrane citrate transporters: the citrate exporter CexA from *A. niger* [68,69], the citrate exporter Cex1 from *Y. lipolytica* [70], and the PkJEN2-2 importer from *Pichia kudriavzevii* [58]; however, the kinetic parameters of these transporters [69,70] were not determined. Regardless the fact that the present work measured the import of carboxylates, the experimental evidence demonstrates that permeases can work both as importers and exporters [9,71]. Additionally, previous reports have indicated that exporters and importers present low and high affinity for substrates, respectively [64,72], so CjSlc5p, CjAto5p, and CjJen6p can be regarded as general carboxylate importers and/or exporters that are capable of recognizing a wide range of substrates (such as mono-, di-, and tricarboxylates).

### 4.3. Phylogenetic Roots of the C. jadinii Carboxylate Transporters

The phylogenetic reconstruction of Ato homologs suggests that CjAto5 is distant from CjAto1-4, ScAto1, and YlGpr1, but it shares the same clade with ScAto3 [52], whose function remains unknown. The analysis of the Jen homologs revealed that CjJen1-4 are in the Jen1-cluster, where the majority of the yeast monocarboxylate transporters are found. As for CjJen5 and CjJen6, they present a closer phylogenetic relationship with succinate transporters DH24 from *D. hansenii* [56], KlJen2 from *K. lactis* [33,48,73], and CaJen2 (a dicarboxylate and sugar acid permease from *C. albicans*) [31,33] than to the succinate transporter DH18 from *D. hansenii* [56], the dicarboxylate and di/tricarboxylate permeases PkJen2-1 and PkJen2-2 from *Pichia kudriavzevii* [58], and the sub-clade containing the clustered six Jen homologs from *Y. lipolytica* [60,65]. Despite being phylogenetically close, the specificity of CjJen5 and CjJen6 was found to be very distinct, suggesting the divergence of CjJen6.

The phylogenetic reconstruction of the *CjSLC5* homologs only identified members of the eukaryotic domain of life, namely ascomycetes and basidiomycetes. A high prevalence of homologs was found in *Fusarium* species, and none was found in *S. cerevisiae*. The majority of SLC5 homologs have been annotated as putative neutral amino acid transporters. This fact may be explained by the existence of two homologs functionally characterized as amino acid transporters: the Mtr/AAP1 from *N. crassa* [61] and PcMtr from *P. chrysogenum* [62] (Figure 9, E1 clade). These data suggest that CjSlc5 may also behave as an amino acid transporter, though no functional studies were made to confirm this hypothesis. Cássio et al. (1993) reported the presence of a facilitated diffusion system in *C. jadinii* that is likely to operate as a general organic permease, accepting mono-, di-, and tricarboxylates as well as amino acids, as glycine, and glutamic acid [25].

### 4.4. Structural Features of the C. jadinii Carboxylate Transporters

To obtain insights into the structural–functional properties of the different transporters, we followed several strategies that included multiple-sequence alignments with characterized carboxylate transporters, molecular docking studies, and analyses of protein pore size variations. The alterations in the highly conserved L93 residue of the signature NPAPLGL(M/S) motif of the AceTr family [10] observed in CjAto5 (M85) may play a relevant role on its broader substrate specificity. Another spot-on residue alteration observed in CjAto5 was found to be precisely located at the narrowest constriction site, namely at L219-ScAto1 where a V210 is found. Recent studies have reported the influence of such alteration into a larger aperture of the pore that enable Ato1 (L219V) to more efficiently transport lactate. In addition, the substitution of ScAto1-L219A allows for the succinate transport [14,15]. Additionally in CjAto5, the substitution of conserved G/A-97 to a serine located in TMS1 near the narrowest constriction site formed by F17, Y72, and L131 residues [12] might influence function and contribute to the increase in the narrowest constriction site in 0.99 Å compared to ScAto1.

In this work, we describe the first AceTr homologs in yeast with affinity for dicarboxylates and tricarboxylates [10]. Our molecular docking analysis uncovered a residue located at the end of TMS5 as a binding site for succinate present in CjAto2 (S211) and CjAto5 (K221). In addition, besides dicarboxylates, CjAto5 also accepts tricarboxylic acids. Based on our analysis, the non-conserved residues Q103 and K230, located in TMS1 and TMS6, respectively, may act as putative binding sites for citrate.

Regarding Jen protein members, our results highlighted two residues in CjJen6 that were not conserved across all Jen members. On TMS1, W154 (in ScJen1) was shown to be substituted for L65 in CjJen6 and also present in the succinate transporters CaJen2 [31,33] and KlJen2 [33,48,73]. In addition, in TMS7, a highly conserved leucine was found to be substituted for valine in residue 260 of CjJen6.

Future studies will be necessary to determine the contribution of these residues to the broader specificity of CjAto5 and CjJen6.

## 5. Conclusions

In the present study, novel *C. jadinii* carboxylate transporters belonging to a total of six transporter families with specificity for short-chain carboxylic acids were uncovered and functionally characterized. Two members from the AceTr family are able to transport succinate, CjAto2, and citrate, CjAto5. This latter is the first AceTr family member described as a broad range carboxylate transporter, accepting mono-, di-, and tricarboxylates. In addition, one member from SHS transporter family (CjJen6) and the two homologs from families SSS (SLC member 5) and DASS (SLC member 13) also showed the ability to transport mono-, di-, and tricarboxylates. To our knowledge, CjSlc5 and CjSlc13 are the first members from these families characterized in yeast as carboxylate transporters.

In the future, it will be important to test if the activity of these membrane transporters could lead to the improved bioproduction of carboxylates. Overall, this work increases the knowledge of yeast plasma membrane transporter proteins that can ultimately lead to development of improved bio-processes with an impact in industrial biotechnology.

## Figures and Tables

**Table 1:** Yeast strains used in this study.

Strain	Genotype	Source
*Cyberlindnera jadinii* DSM 2361	Type strain DSM 2361	DSM collection
*Saccharomyces cerevisiae* W303-1A	MATα *ade2 leu2 his3 trp1 ura3*	[29]
*S. cerevisiae jen1*Δ *ato1*Δ	W303-1A; *JEN1::KanMX4 ATO1::HphMX4*	[27]
*S. cerevisiae* CEN.PK113-7D	MATα *URA3 TRP1 LEU2 HIS3*	[30]
IMX1000 (parental strain CEN.PK113-7D)	*MAT*a *ura3-52 trp1-289 leu2-3112 his3*Δ *can1*Δ::*cas9-natNT2 mch1*Δ *mch2*Δ *mch5*Δ *aqy1*Δ *itr1*Δ *pdr12*Δ *mch3*Δ *mch4*Δ *yil166c*Δ *hxt1*Δ *jen1*Δ *ato1*Δ *aqr1*Δ *thi73*Δ *fps1*Δ *aqy2*Δ *yll053c*Δ *ato2*Δ *ato3*Δ *aqy3*Δ *tpo2*Δ *yro2*Δ *azr1*Δ *yhl008c*Δ *tpo3*Δ	[28]
*S. cerevisiae jen1*Δ *ato1*Δ p416GPD	*jen1*Δ *ato1*Δ transformed with p416GPD	[27]
*S. cerevisiae jen1*Δ *ato1*Δ pCaJen2	*jen1*Δ *ato1*Δ transformed with pCaJen2	[31]
*S. cerevisiae jen1*Δ *ato1*Δ pScAto1	*jen1*Δ *ato1*Δ transformed with pScAto1	[10]
*S. cerevisiae jen1*Δ *ato1*Δ pScJen1-GFP	*jen1*Δ *ato1*Δ transformed with pScJen1-GFP	[32]
*S. cerevisiae jen1*Δ *ato1*Δ pCjAto1	*jen1*Δ *ato1*Δ transformed with pCjAto1	This work
*S. cerevisiae jen1*Δ *ato1*Δ pCjAto2	*jen1*Δ *ato1*Δ transformed with pCjAto2	This work
*S. cerevisiae jen1*Δ *ato1*Δ pCjAto3	*jen1*Δ *ato1*Δ transformed with pCjAto3	This work
*S. cerevisiae jen1*Δ *ato1*Δ pCjAto4	*jen1*Δ *ato1*Δ transformed with pCjAto4	This work
*S. cerevisiae jen1*Δ *ato1*Δ pCjJen1	*jen1*Δ *ato1*Δ transformed with pCjJen1	This work
*S. cerevisiae jen1*Δ *ato1*Δ pCjJen2	*jen1*Δ *ato1*Δ transformed with pCjJen2	This work
*S. cerevisiae jen1*Δ *ato1*Δ pCjJen3	*jen1*Δ *ato1*Δ transformed with pCjJen3	This work
*S. cerevisiae jen1*Δ *ato1*Δ pCjJen4	*jen1*Δ *ato1*Δ transformed with pCjJen4	This work
*S. cerevisiae jen1*Δ *ato1*Δ pCjJen5	*jen1*Δ *ato1*Δ transformed with pCjJen5	This work
*S. cerevisiae jen1*Δ *ato1*Δ pCjJen6	*jen1*Δ *ato1*Δ transformed with pCjJen6	This work
IMX1000 pCjAto1	IMX1000 transformed with pCjAto1	This work
IMX1000 pCjAto2	IMX1000 transformed with pCjAto2	This work
IMX1000 pCjAto3	IMX1000 transformed with pCjAto3	This work
IMX1000 pCjAto4	IMX1000 transformed with pCjAto4	This work
IMX1000 pCjAto5	IMX1000 transformed with pCjAto5	This work
IMX1000 pCjJen1	IMX1000 transformed with pCjJen1	This work
IMX1000 pCjJen2	IMX1000 transformed with pCjJen2	This work
IMX1000 pCjJen3	IMX1000 transformed with pCjJen3	This work
IMX1000 pCjJen4	IMX1000 transformed with pCjJen4	This work
IMX1000 pCjJen5	IMX1000 transformed with pCjJen5	This work
IMX1000 pCjJen6	IMX1000 transformed with pCjJen6	This work
IMX1000 pCjSlc16	IMX1000 transformed with pCjSlc16	This work
IMX1000 pCjSlc5	IMX1000 transformed with pCjSlc5	This work
IMX1000 pCjTDT	IMX1000 transformed with pCjTDT	This work
IMX1000 pCjSlc13-1	IMX1000 transformed with pCjSlc13-1	This work
IMX1000 pCjSlc13-2	IMX1000 transformed with pCjSlc13-2	This work

**Table 2:** Plasmids used in this study.

Plasmid	Description	Reference
p416GPD	Glyceraldehyde-3-phosphate dehydrogenase (GPD) promoter; *URA3* marker	[34]
pJen1-GFP	*ScJen1* cloned in p416 under the control of GPD promoter with the GFP gene	[32]
pCaJen2	*CaJen2* cloned in p416 under the control of GPD promoter	[31]
pScAto1	*ScAto1* cloned in p416 under the control of GPD promoter	[10]
pCjAto1	*CEP24587* cloned in p416 under the control of GPD promoter	This work
pCjAto2	*CEP20823* cloned in p416 under the control of GPD promoter	This work
pCjAto3	*CEP20690* cloned in p416 under the control of GPD promoter	This work
pCjAto4	*CEP20822* cloned in p416 under the control of GPD promoter	This work
pCjJen1	*CEP23088.1* cloned in p416 under the control of GPD promoter	This work
pCjJen2	*CEP21966.1* cloned in p416 under the control of GPD promoter	This work
pCjJen3	*CEP22358.1* cloned in p416 under the control of GPD promoter	This work
pCjJen4	*CEP21989.1* cloned in p416 under the control of GPD promoter	This work
pCjJen5	*CEP21602.1* cloned in p416 under the control of GPD promoter	This work
pCjJen6	*CEP25129.1* cloned in p416 under the control of GPD promoter	This work
pCjSlc16	XP_020067635.1 cloned in p416 under the control of GPD promoter	This work
pCjSlc5	XP_020068154.1 cloned in p416 under the control of GPD promoter	This work
pCjTDT	XP_020068891.1 cloned in p416 under the control of GPD promoter	This work
pCjSlc13-1	XP_020069270.1 cloned in p416 under the control of GPD promoter	This work
pCjSlc13-2	XP_020073044.1 cloned in p416 under the control of GPD promoter	This work
pCjAto5	XP_020067765.1 cloned in p416 under the control of GPD promoter	This work

**Table 3:** Kinetic parameters for ^14^C-acetic acid (pH 6.0) transport estimated in the cells of *S. cerevisiae* W303-1A *jen1*Δ *ato1*Δ expressing the *CjATO1*, *CjATO3*, *CjATO4*, and *CjJEN1-5* proteins. Cells were grown on glucose until the mid-exponential growth phase and shifted to YNB acetate (0.5%, pH 6.0) for 6 h.

Transporter	*K*_m_ (mM)	*V*_max_ (nmol s^−1^ Acetate mg^−1^ Dry wt.)
CjATO1	12.18 ± 4.67	4.92 ± 1.32
CjATO3	9.17 ± 4.70	3.85 ± 1.23
CjATO4	1.28 ± 0.38	0.69 ± 0.10
CjJEN1	2.15 ± 0.60	0.86 ± 0.12
CjJEN2	1.26 ± 0.35	0.49 ± 0.06
CjJEN3	3.21 ± 1.07	1.34 ± 0.25
CjJEN4	1.62 ± 0.43	0.62 ± 0.08
CjJEN5	5.18 ± 1.51	2.08 ± 0.39

## Data Availability

Not applicable.

## Supplementary Materials

The following are available online at https://www.mdpi.com/article/10.3390/jof8010051/s1. Figure S1: Multiple sequence alignment of Ato homologs; Figure S2: Molecular docking of *Escherichia coli* Satp 3D model; Figure S3: Hydrophobic constriction site analyzed in ScAto1, CjAto2, CjAto5, and CjAto6; Figure S4: Multiple sequence alignment of Jen homologs; Figure S5: Detailed ATO phylogenetic tree; Figure S6: Detailed JEN phylogenetic tree; Figure S7: Detailed SLC5 phylogenetic tree; Table S1. Homology parameters of *C. jadinii* carboxylate transporter homologs from distinct transporter families; Table S2. Oligonucleotides used for strain construction, cloning, and expression; Table S3. Parameters obtained with HHPred for 3D model construction; Tables S4 and S5: Docking parameters and data from the molecular docking studies with Ato homologs.
