# Supplementary material for: Uncovering Novel Plasma Membrane Carboxylate Transporters in the Yeast Cyberlindnera jadinii
Source: J Fungi (Basel). 2022 Jan 5;8(1):51. doi: 10.3390/jof8010051 (PMC8779868; doi:10.3390/jof8010051)
Supplement: Supplementary file 1 [file jof-08-00051-s001.zip › jof-1516984-supplementary.pdf]

Uncovering novel plasma membrane carboxylate transporters in the yeast *Cyberlindnera jadinii*

Sousa-Silva, M. [1,2], Soares, P. [1,2], Alves, J. [1,2], Vieira, D. [1,2], Casal, M. [1,2], Soares-Silva, I. [1,2*]

[1]Institute of Science and Innovation for Bio-Sustainability (IB-S), University of Minho, Portugal

[2]Centre of Molecular and Environmental Biology (CBMA), Department of Biology, University of Minho, Campus de Gualtar, 4710-057 Braga, Portugal

*Corresponding author: Isabel Soares-Silva; ijoao@bio.uminho.pt

Supplementary material

Table S1. Identification of *C. jadinii* carboxylate transporter homologs of the distinct transporter families. For ScAto1 and ScJen1 homologs, homology search was performed by BlastP. Protein conserved domains were determined in the Conserved Domain Database (CDD) and the number of transmembrane segments (TMSs) was determined with TMHMM Server v.2.0.

Accession number	Query	Query cover	Identity	E-value	TMSs	ID gene
**XP_020070445.1**	NP_009936.1 Ady2p [*S. cerevisiae*]	98 %	56 %	$2e^{-99}$	4	CjAto1
**XP_020073179.1**		96 %	57 %	$2e^{-96}$	6	CjAto2
**XP_020073031.1**		91 %	55 %	$2e^{-100}$	5	CjAto3
**XP_020073178.1**		98 %	57 %	$4e^{-109}$	5	CjAto4
**XP_020067765.1**		94 %	41 %	$6e^{-72}$	6	CjAto5
**CEP23088.1**	NP_012705.1 Jen1p [*S. cerevisiae*]	82 %	58 %	0	10	CjJen1
**CEP21966.1**		85 %	51 %	$1e^{-175}$	11	CjJen2
**CEP22358.1**		77 %	40 %	$1e^{-119}$	12	CjJen3
**CEP21989.1**		76 %	41 %	$5e^{-116}$	12	CjJen4
**CEP21602.1**		67 %	43 %	$1e^{-103}$	10	CjJen5
**CEP25129.1**		82 %	41 %	$8e^{-127}$	12	CjJen6

Accession number	Domain designation	Accession	Description	E-value	TMSs	ID gene
**XP_020067635.1**	MFS_MCT_SLC16	cd17352	Monocarboxylate transporter (MCT) family of the Major Facilitator Superfamily of transporters	$2.27e^{-64}$	12	CjSlc16
**XP_020068154.1**	SLC5-6-like_sbd super family	cl00456	Solute carrier families 5 and 6-like; solute binding domain	$3.25e^{-14}$	11	CjSlc5
**XP_020068891.1**	TDT super family	cl04176	Tellurite-resistance/Dicarboxylate Transporter (TDT) family	$1.19e^{-83}$	9	CjTDT
**XP_020069270.1**	SLC13_permease	cd01115	Permease SLC13 (solute carrier 13); Sodium/dicarboxylate cotransporter NaDC-1	$1.56e^{-98}$	12	CjSlc13-1
**XP_020073044.1**			Permease SLC13 (solute carrier 13); Sodium/dicarboxylate cotransporter NaDC-1	$7.26e^{-103}$	12	CjSlc13-2

Table S2. Oligonucleotides used for strain construction, cloning and expression.

Name	Sequence
**p416-CJAd1_fwd**	GCAGGATCCATGTCAGACAAGGAAAGC
**p416-CJAd1_rev**	GCAGAATTCCTAGGAGTGCACTTGAGC
**p416-CJAd2_fwd**	GCAGGATCCATGTCTAGTATTAATGAG
**p416-CJAd2_rev**	GCAGAATTCTCACAAATGGCTAGCACC
**p416-CJAd3_fwd**	GCAGGATCCATGGCCGCCAACGTTGAC
**p416-CJAd3_rev**	GCAGAATTCTCAGGCACGCTTTGCACC
**p416-CJAd4_fwd**	GCAGGATCCATGTCGGACAAGGAAAAC
**p416-CJAd4_rev**	GCAGAATTCTCAAGAACGCTTTGCACC
**p416-CJJen1_fwd**	GCA GGATCCATGCACAAACTTGAAGAG
**p416-CJJen1_rev**	GCAGAATTCTCACTTCTTCTCCTGTGG
**p416-CJJen2-new_Fwd**	GCAGGATCCATGACTTCACCATTGCCT
**p416-CJJen2-new_Rev**	GCACTCGAGTCACTCACTTGAAGAGCC
**p416-CJJen3_fwd**	GCAGAATTCATGGCAATGTCTGATGTT
**p416-CJJen3_rev**	GCAGAATTCTCAGGTTTTTTCATTATG
**p416-CJJen4-new_Fwd**	GCAGGATCC ATGACTGCTGGGAGATAC
**p416-CJJen4-new_Rev**	GCACTCGAGCTAGTCTCTAGCAGATTC
**p416-CJJen5_fwd**	GCAGGATCCATGGACTGGGATGCTTTC
**p416-CJJen5_rev**	GCAGAATTCTCACTTTGGCTCTATCTT
**Cut-Jen6_Fwd**	GCCTCTAGAATGGGATTCAAGACGTAC
**Cut-Jen6_Rev**	GCCGAATTCTCATTTAACCTCAGAAAC
**ct1.635_Fwd**	GCCACTAGTATGACTGAAATCATCACT
**ct1.635_Rev**	GCCGTCGACTCAGAATTTACAAATTCT
**ct2.154_Fwd**	GCCACTAGTATGTTTGCAGAGACCGAG
**ct2.154_Rev**	GCCGTCGACTCACGAGTTGTCAGCACA
**ct3.891_Fwd**	GCCACTAGTATGACATCTGATGAGAAT
**ct3.891_Rev**	CCGTCGACTCAATTCCTTTCACTGTT
**ct4.270_Fwd**	GCCACTAGTATGAAATTCTCCCTCTCT
**ct4.270_Rev**	GCCGTCGACTTAACCGTGTAAAGTTGC
**ct8.044_fwd**	GCCACTAGTATGAAGTTCTCCCATTCG
**ct8.044_rev**	GCCCTCGAGTCACATCCCTGTTAATCT
**ct9.7765_fwd**	GCCACTAGTATGTCGACCTCTTCTCTC
**ct9.7765_rev**	GCCGAATTCCTAAACTCTTGGTGCATG

Table S3. Parameters obtained with HHPred for 3D-model construction.

Protein templates	PDB Hit	Organism	E-value	Similarity	Identity	Score	Probability (%)
**ScAto1**	5YS3	*Citrobacter koseri*	$3.7e^{-32}$	0.566	35%	236.5	100.0
**EcSatP**	5YS3	*Citrobacter koseri*	$1.5e^{-36}$	1.498	92%	235.4	100.0
**CjAto2**	5YS3	*Citrobacter koseri*	$6.7e^{-33}$	0.538	32%	237.2	100.0
**CjAto5**	5YS3	*Citrobacter koseri*	$7e^{-35}$	0.525	26%	252.0	100.0

Table S4. Residues of Ato homologs presenting strong intramolecular interactions with lactate, succinate and citrate identified by molecular docking studies.

3D-Protein templates	Lactate	Succinate		Citrate
	(-1)	*(-1)*	*(-2)*	*(-3)*
**ScATO1**	R111; T238; N179; T222; T102; S106; E140; T209; N255	T238; N179; R111; T222; T102; Q133; S208; N145; N255; T209; E140	R111; N179; T238; T222; T209; S208; N145	N255; T209; S208; N89; N145; Q133; T238; H230; R111; N179
**CjATO2**	K244; S189; N127; N91; T203; I97; T99; Y158	N236; K244; S189; C185; T203; N91; T84; T99; D150; R214; Q219; S211; W144; Y158	S189; K244; N127; T203; W144; T99; Q219; R214; Y158	*ND*
**CjATO5**	N101; G220; Q103; T229; K230	E125; K221	K221	N137; S199; K198; N81; K221; Q103; T229; K230; G220

Note: *ND* - not determined.

Table S5. Average of the binding affinity values [kcal/mol] calculated with PyRx software for the docking of Ato proteins with the distinct charged substrates tested.

3D-Protein templates	Average of binding affinities (kcal/mol) at different binding sites						
	**Lactate (-1)**						
	**S4**			**S3**	**S2**	**S1**	
	**a**	**b**	**c**			**a**	**b**
**ScAto1**	-	-3.8	-	-4.1	-3.9	-3.3	-3.1
**EcSatP**	-3	-	-	-3.6	-3.8	-3	-
**CjAto2**	-3.4	-	-3.4	-4.2	-2.6	-3.4	-3.1
**CjAto5**	-3.2	-3.1	-	-3.3	-3.8	-3.4	-
	**Succinate (-1)**						
	**S4**			**S3**	**S2**	**S1**	
	**a**	**b**	**c**			**a**	**b**
**ScAto1**	-	-4.0	-	-5	-4.5	-3.9	-3.8

**EcSatP**	-3.6	-	-	-4.5	-4.6	-4.2	-
**CjAto2**	-4.2	-	-4.2	-4.7	-3.2	-3.9	-4
**CjAto5**	-3.8	-	-	-3.9	**-4.7**	-	-
	**Succinate (-2)**						
	**S4**			**S3**	**S2**	**S1**	
	**a**	**b**	**c**			**a**	**b**
**ScAto1**	-	-4.0	-	-5	-4.4	-3.7	-
**EcSatP**	-3.4	-	-	-4.4	**-4.7**	-4.3	-
**CjAto2**	-4	-	-4.1	-4.8	-3.1	**-4.1**	-4
**CjAto5**	-3.8	-	-	-3.9	**-4.6**	-	-
	**Citrate (-3)**						
	**S4**			**S3**	**S2**	**S1**	
	**a**	**b**	**c**			**a**	**b**
**ScAto1**	-	-5.0	-	-6.1	-3.7	-4.6	-
**EcSatP**	-4.2	-	-	-5	**-4.3**	-4.2	-
**CjAto5**	-4	-4	-	-3.5	**-5.5**	-4.5	-4.2

Figure S1. Multiple sequence alignment of Ato homologs from *Saccharomyces cerevisiae* (Ato1, Ato2, Ato3), *C. jadinii* (CjAto1-5) and the *Escherichia coli* SatP. The sequence alignment was built with ClustalOmega (https://www.ebi.ac.uk/Tools/msa/clustalo/). Localization of transmembrane segments (TMSs) was predicted by the PSI/TM-Coffee software (http://tcoffee.crg.cat/apps/tcoffee/do:tmcoffee). Grey background highlights the previously identified and characterized signature motifs of the AceTr family (Ribas et al., 2019). Blue rectangles indicate residues from the narrowest hydrophobic constriction site F98-Y155-L219 (refer to ScAto1p) (Qiu et al., 2018).

```
                                             1stTMS
ScAto1      GRQKFLKSDLYQAFG-GTLNPGLAP-APVHKFANPAPLGLSAFALTTFVLSMFNARAQGI  115
ScAto2      GRQKFLRDDLFEAFG-GTLNPGLAP-APVHKFANPAPLGLSGFALTTFVLSMFNARAQGI  114
ScAto3      GSSTYRRRDLLNALDRGDGEEGNCAKYTPHQFANPVPLGLASFSLSCLVLSLINANVRGV  112
CjAto1      GRTKVLRSELWNAFG-GDLQPGIHA-PPPRRFANPAPLGLCGFALTTFVLSMSNARAMGI  110
CjAto2      GRMKVRKSELWSAFG-GDLQPGVHA-QPQRKFANPVPIGLCGFALTTLVLSMANARAMGI  97
CjAto3      GRTKVLRSELWNAFG-GDLQPGIHA-TPPRRFANPAPLGLCAFALTTFVLSMVNARAMGI  104
CjAto4      GHQKVLRSELWTAFG-GDLQPGVHA-PPPQRLANPAPLGLCGFALTTFVLSMANARAMGV  106
CjAto5      GNQAFSKKDLFNAFA-GDLQPGLHA-TPHRPMGNPVPMGLTSFCICCFVVSLVNAQARGV  107
EcSatp      --------------------------MGNTKLANPAPLGLMGFGMTTILLNLHNVGYFA-  33
                                           :.**.*:** .* :  :::.: *.   .
               2ndTMS                      3rdTMS
ScAto1      TVPNVVVGCAMFYGGLVQLIAGIWEIALENTFGGTALCSYGGFWLSFAAIYI-PWFGILE  174
ScAto2      TIPNVVVGCAMFYGGLVQLIAGIWEIALENTFGGTALCSFGGFWLSFGAIYI-PWFGILD  173
ScAto3      TDGKWALSLFMFFGGAIELFAGLLCFVIGDTYAMTVFSSFGGFWICYGYGLT-DTDNLVS  171
CjAto1      TVANVAVGPAFFYGGIIQLLSGMWEISLDNTFGGTVLSSYGGFWLSWAAIQI-DWFGIQR  169
CjAto2      RTPNVAVAPAFFYGGFAQILAGMWEIALENTFGSVVLTSYGCFWLSWAAIEI-DWFGIKA  156
CjAto3      TTPNIVVGLALFYGGFVQLLAGMWEIALDNTFGGTALSSYGGFWMSYAAIQI-DWFGIKS  163
CjAto4      TIPNAAVGAACFYGGLVQLLAGMWEISLDNTFGGTALSSYGGFWMSWAAIQI-DWFGIKK  165
CjAto5      TNAKVIASCALFFAGVVETISGLWCLVIENTFAATALGSFGGFWMGYAGLLI-DAFGITS  166
EcSatp      -LDGIILAMGIFYGGIAQIFAGLLEYKKGNTFGLTAFTSYGSFWLTLVAILLMPKLGLTD  92
                   .   *:.*  : ::*:      :*:. ..: *:* **:           .:
                      4thTMS                   5thTMS
ScAto1      AYEDNESDLNNALGFYLLGWAIFTFGLTVCTMKSTVMFFLLFFLLALTFLLLSIGHFANR  234
ScAto2      AYKDKESDLGNALGFYLLGWALFTFGLSVCTMKSTIMFFALFFLLAVTFLLLSIANFTGE  233
ScAto3      GYTDP-TMLNNVIGFFLAGWTVFTFLMLMCTLKSTWGLFLLLTFLDLTFLLLCIGTFIDN  230
CjAto1      AYDDP-IMLNNGLGFFLLGWVIFTLMVLICTVKSTVAFFSLFFFLEMTFLLLTIGEFTRS  228
CjAto2      AYDDP-IELENAIGFFLLGWVIFTFLILLCTMKSTVAFFSMFFLLEITFILLTVASFTRH  215
CjAto3      AYTDP-IELANAVSFFLLGWTIFTFMILLCTVKSTVSFFSLFFFLEITFLLLTIGDFTRR  222
CjAto4      AYDDP-IMLANAVGFFLLGWTIFTFMLVLCTVKSTVAFFSLFFFLDITFLLLTIGEFTRK  224
CjAto5      SYSTT-EELGNALGFYLTAWTIFAFLMWLCTFKSTWPFFILFLLVWVFLMCLAIGKYNDN  225
EcSatp      A------PNAQFLGVYLGLWGVFTLFMFFGTLKGARVLQFVFFSLTVLFALLAIGNIAGN  146
            .         : :..:*  * :*:: : . *.*.:  :  ::  : : :  * :.
                6thTMS
ScAto1      LGVTRAGGVLGVVVAFIAWYNAYAGVATKQNSYVLARPFPLPSTERVIF---  283
ScAto2      VGVTRAGGVLGVIVAFIAWYNAYAGIATRQNSYIMVHPFALPSNDKVFF---  282
ScAto3      NNLKMAGGYFGILSSCCGWYSLYCSVVSPSNSYLAFRAHTMPNAP-------  275
CjAto1      VGVTRAAGVFGVITSFLGWYNALAGFATRENSYFVATAVPLPGAKRAQVHS-  279
CjAto2      VGCQRAGGVFGVITGFLAWYNAYAGIATKEISYFVPKPWPLPGASHL-----  262
CjAto3      VGVTRAGGVFGVITAFIAWYNAFAGIATKENSYITIKAWPLPGAKRA-----  269
CjAto4      TGVSRAGGVFGVITSFIAWYNAFAGLATKENSYVVAIPLPLPGAKRS-----  271
CjAto5      TTATKAGGVLGLVATFVGFFIVYAGVADSSNSYLTIPASPMPHAPRV-----  272
EcSatp      AAIIHFAGWIGLICGASAIYLAMGEVLNEQFGRTVL---PIGESH-------  188
                  .* :*::    . :     .   . .        :
```

Figure S2. Molecular docking of *Escherichia coli* Satp 3D-model, based on SatP_Ck structure, with the substrates lactate (blue ligand), succinate (orange ligand) and citrate (green ligand). The four binding sites are depicted from S1 to S4 sites, including the localization of N- and C-terminal of the protein. The narrowest hydrophobic constriction of the anion pathway formed by F17, Y72 and L131 is also represented.

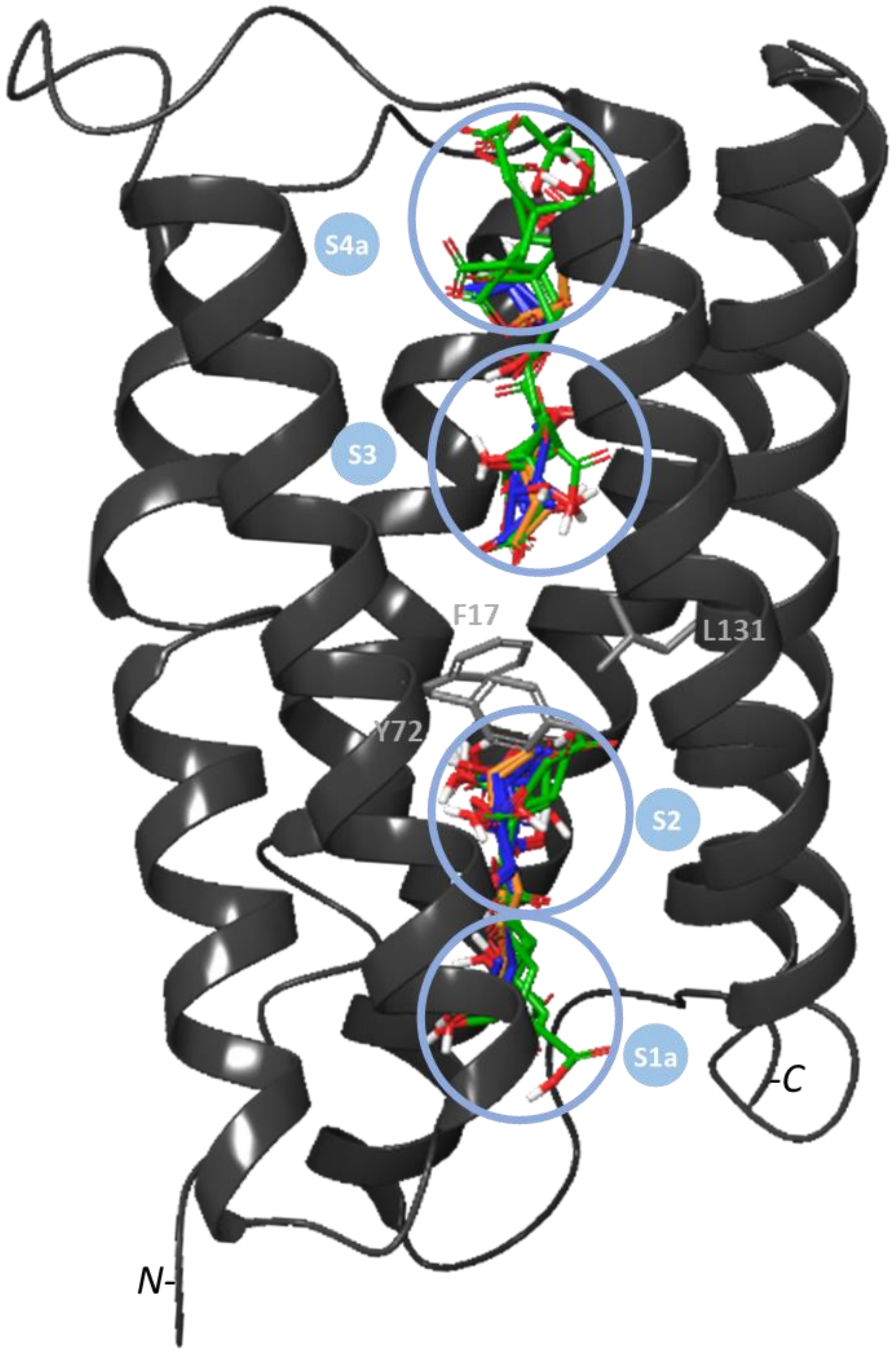

Figure S3. Predicted 3D structures of Ato proteins visualized with Maestro and HOLE softwares. On the left are the pore predictions: blue (larger aperture), green (intermediate pore size) and red (constricted pore). The top view of the narrowest hydrophobic constriction site of the anion pathway is also presented, including the predicted distances (Å) between represented residues.

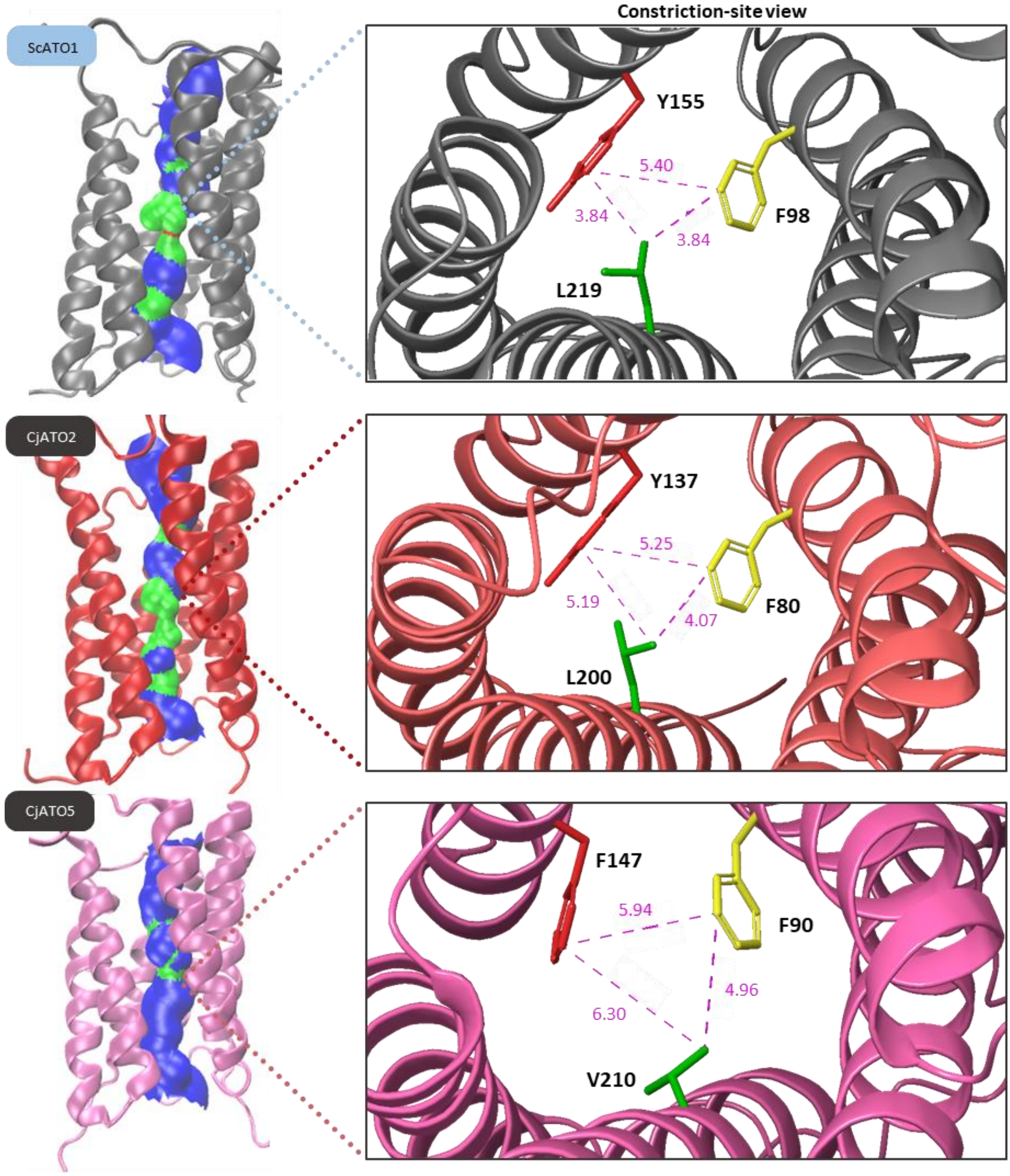

Figure S4. Multiple sequence alignment of Jen homologs from *Saccharomyces cerevisiae*, *Candida albicans* (CaJen1-2), *Kluyveromyces lactis* (KlJen1-2) and C. jadinii (CjJen1-6). The sequence alignment was built with ClustalOmega (https://www.ebi.ac.uk/Tools/msa/clustalo/) and the localization of transmembrane segments (TMSs) predicted by the PSI/TM-Coffee (http://tcoffee.crg.cat/apps/tcoffee/do:tmcoffee). Grey background highlights the previously identified and characterized motifs of the SHS family for carboxylate transporter affinity (Soares-Silva et al., 2007; 2011).

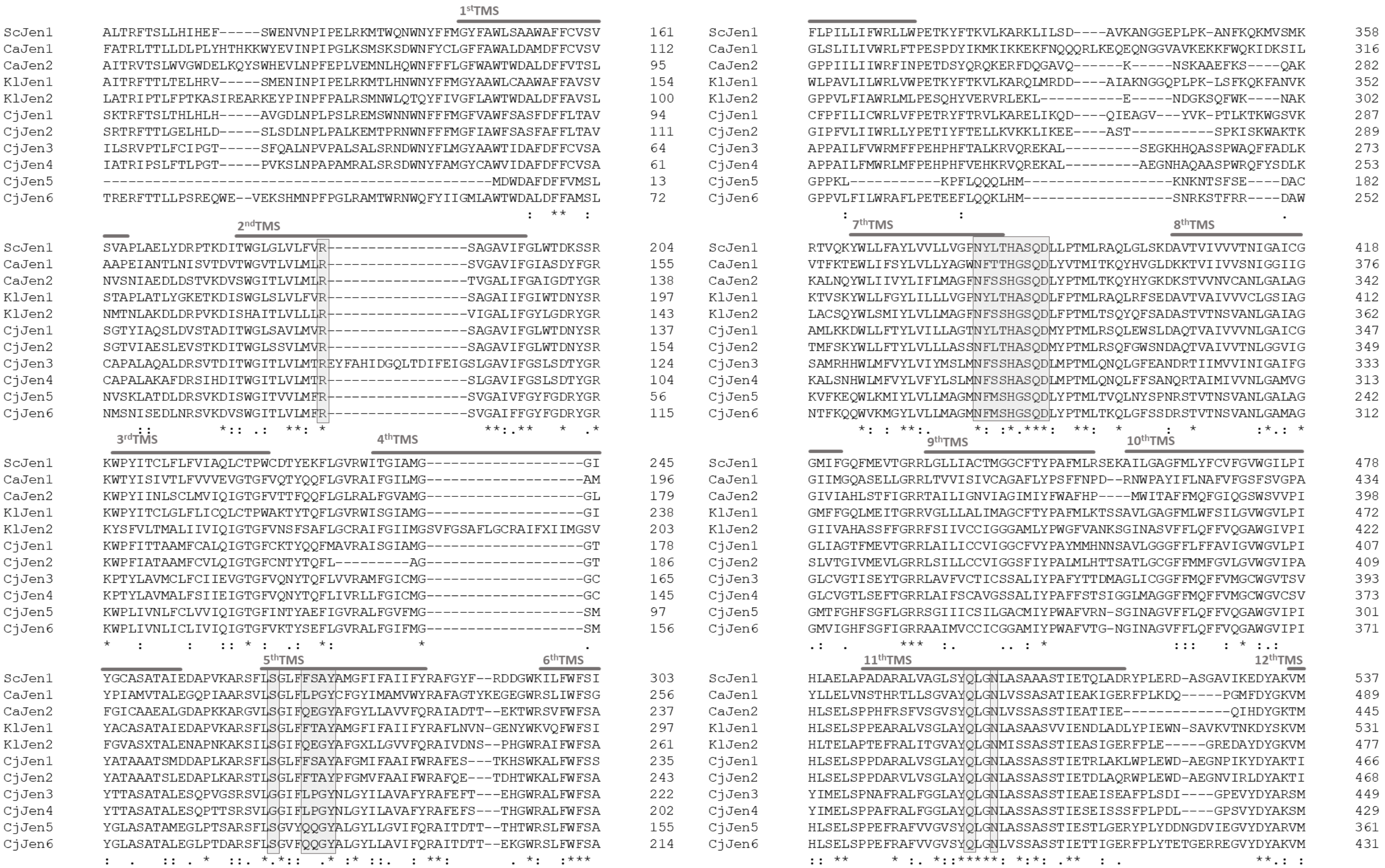

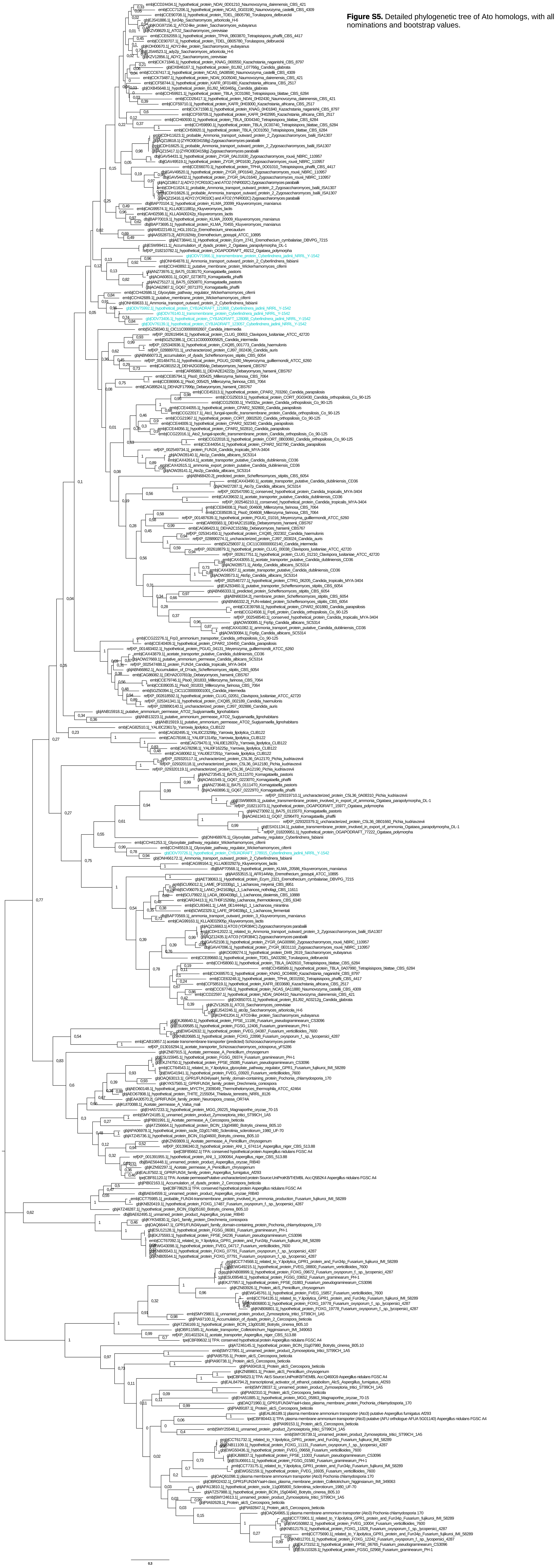

Figure S5. Detailed phylogenetic tree of Ato homologs, with all nominations and bootstrap values.

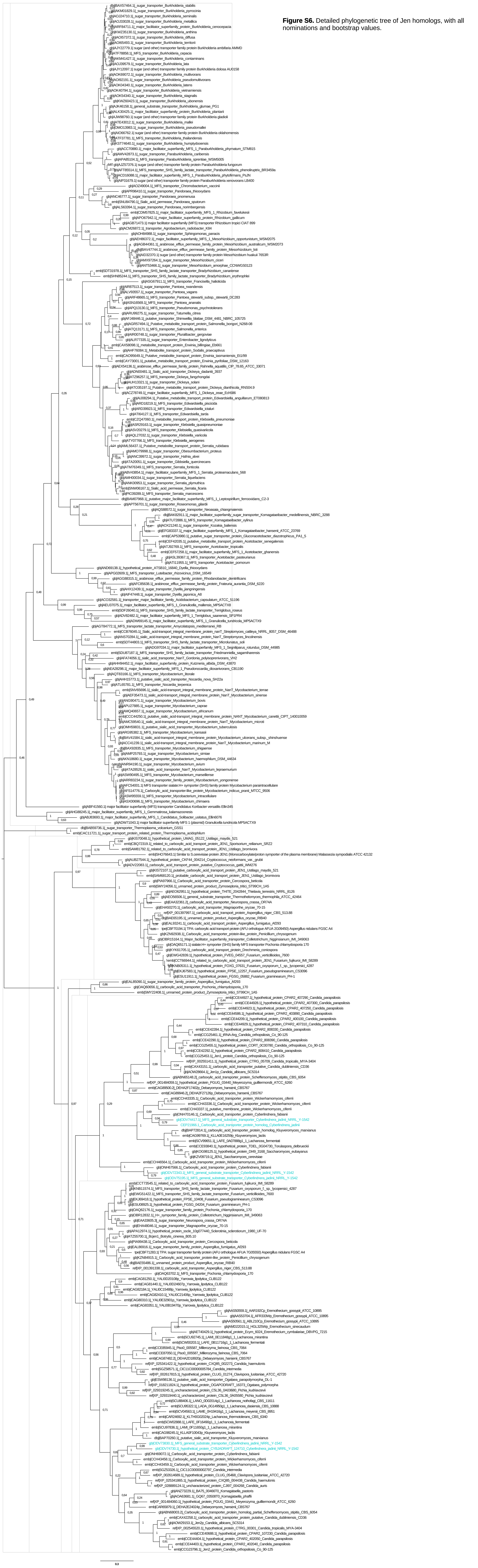

Figure S6. Detailed phylogenetic tree of Jen homologs, with all nominations and bootstrap values.

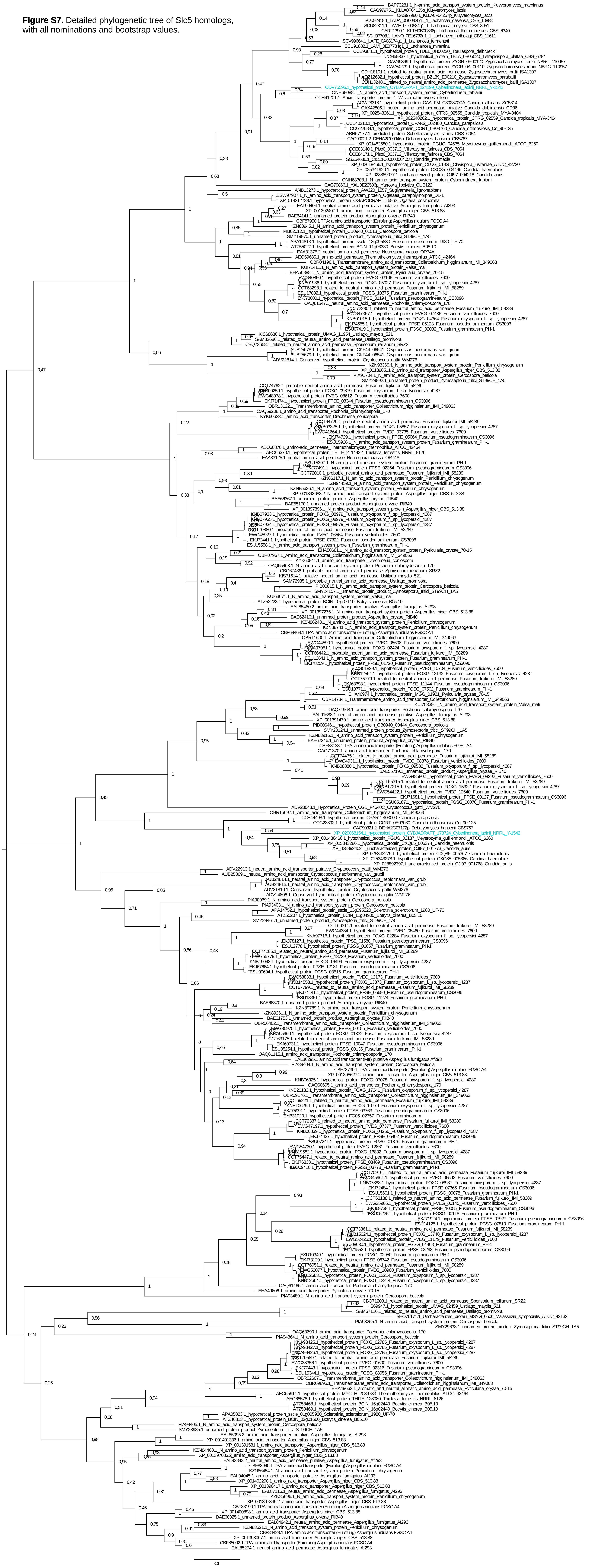

Figure S7. Detailed phylogenetic tree of Slc5 homologs, with all nominations and bootstrap values.
